# Dairy Consumption and Risk of Cardiometabolic Diseases: A Prospective Cohort Study of the China Kadoorie Biobank

**DOI:** 10.1016/j.tjnut.2026.101388

**Published:** 2026-01-31

**Authors:** Maria G Kakkoura, Hao Wang, Andri Iona, Yiping Chen, Christiana Kartsonaki, Pek Kei Im, Iona Y Millwood, Keren Papier, Canqing Yu, Pei Pei, Dianjianyi Sun, Ling Yang, Daniel Avery, Xiaoming Yang, Min Yu, Jun Lv, Junshi Chen, Liming Li, Zhengming Chen, Huaidong Du, China Kadoorie Biobank Collaborative Group

**Affiliations:** 1 Clinical Trial Service Unit and Epidemiological Studies Unit, Nuffield Department of Population Health, University of Oxford, Oxford, United Kingdom; 2 NCDs Prevention and Control Department, Zhejiang CDC, Zhejiang, China; 3 Cancer Epidemiology Unit, Nuffield Department of Population Health, University of Oxford, Oxford, United Kingdom; 4 Department of Epidemiology and Biostatistics, School of Public Health, Peking University, Beijing, China; 5 Peking University Center for Public Health and Epidemic Preparedness and Response, Beijing, China; 6 Key Laboratory of Epidemiology of Major Diseases (Peking University), Ministry of Education, Beijing, China; 7 China National Center for Food Safety Risk Assessment, Beijing, China

**Keywords:** dairy products, China Kadoorie Biobank Study, cardiovascular disease, diabetes, metabolomic markers

## Abstract

**Background:**

Previous evidence on the associations of dairy intake with risk of cardiometabolic diseases has been inconsistent with studies showing inverse, null, or positive associations.

**Objectives:**

We aimed to assess these associations in China, where dairy consumption level is low and cardiometabolic disease patterns differ from those in the West.

**Methods:**

The China Kadoorie Biobank is a prospective cohort study with ∼512,000 adult participants recruited from 10 diverse localities in China during 2004–2008. At baseline and periodic resurveys, information on the consumption frequency of major food groups was collected using a validated interviewer-administered laptop-based questionnaire. During ∼ 5.4 million person-years of follow-up, 18,306 diabetes, 33,946 ischemic heart diseases [IHD, including 3888 acute myocardial infarction (MI)], 33,670 ischemic stroke, 7191 intracerebral hemorrhage (ICH) cases, and 13,241 cardiovascular deaths were recorded. Cox regression was used to calculate adjusted hazard ratios (HRs) relating dairy intake to cardiometabolic disease risk.

**Results:**

At baseline, 10.7% of participants regularly consumed (i.e., ≥4 d/wk) dairy products, whereas 70.0% reported never or rare consumption. After adjusting for potential confounders including body mass index, dairy consumption was significantly and positively associated with IHD but inversely associated with risks of acute MI, ICH and cardiovascular death, with HRs for regular consumers compared with nonconsumers being 1.09 (95% CI: 1.06, 1.12), 0.88 (0.80, 0.98), 0.69 (0.62, 0.76), and 0.82 (0.77, 0.87), respectively, but not with diabetes and IS. These associations were largely independent of systolic blood pressure.

**Conclusions:**

In Chinese adults, higher dairy consumption was associated with lower risks of acute MI, ICH, and cardiovascular death. Future studies are warranted to further elucidate these relationships and their causality.

## Introduction

Cardiometabolic diseases remain one of the leading causes of morbidity and mortality globally, encompassing a range of conditions such as diabetes, ischemic heart disease (IHD), and stroke [1]. In China alone, the burden of cardiometabolic diseases is substantial, with >3 million deaths diagnosed annually being attributed to these conditions [2]. Although factors such as hypertension, obesity, dyslipidemia, and raised blood glucose are well-established risk factors for cardiometabolic diseases, emerging evidence suggests that dietary habits, including dairy intake, may also play a role [3].

Numerous prospective studies and meta-analyses, primarily conducted in Western populations, have explored the relationship between dairy intake and risk of cardiometabolic diseases, yielding mixed findings: some suggest an inverse association between dairy intake and risk of diabetes [4,5], IHD, and stroke [[5], [6], [7], [8]], whereas others indicate no significant association [7,9,10] or even a positive association with certain cardiovascular disease (CVD) risks [7,11]. The complexity of dairy's impact on cardiometabolic health may be attributed to its heterogeneity in the dairy matrix and diverse nutritional composition, including SFAs, vitamins, minerals such as calcium, magnesium, potassium, bioactive peptides and probiotics, each potentially exerting different effects on cardiovascular physiology [6,12].

However, research on large prospective studies on the associations of dairy intake with incident cardiometabolic diseases in non-Western populations remains very limited. Disparities in dietary habits, types of dairy products consumed [13,14], and genetic factors influencing dairy metabolism between Western and other populations [15] underscore the need for research in diverse populations. In China, for example, despite an increase in per capita milk supply over recent decades [16], the mean dairy milk consumption remains significantly lower than the recommended 300 mL/d intake by the 2022 Chinese dietary guidelines [17], with distinctive dairy consumption patterns that include extremely low cheese intake [18] and highly prevalent lactase nonpersistence [15] (>99%). In addition, the higher incidence of certain cardiometabolic diseases, particularly of intracerebral hemorrhage (ICH), in China than in the West [19] may provide a higher statistical power to these studies and therefore help to clarify the associations of dairy consumption with these diseases as well as their subtypes.

Therefore, we examined the associations between habitual dairy consumption and the incidence of major cardiometabolic outcomes, including diabetes, IHD, acute myocardial infarction (MI), ischemic stroke (IS), ICH, and cardiovascular death in the China Kadoorie Biobank (CKB) study, a comprehensive nationwide prospective cohort study of Chinese adults. Additionally, we explored the associations of dairy intake with cardiometabolic disease-related traits (including adiposity and blood pressure) and biomarkers to elucidate the potential mechanisms linking dairy intake and cardiometabolic diseases. Potential modifying effects of sociodemographic factors and other lifestyle factors on observed associations were also examined.

## Methods

### Study design and population

The CKB is a population-based prospective study of over half a million adults recruited from 10 diverse regions (5 urban and 5 rural) across China. As described elsewhere [20], the study design aimed at capturing a broad spectrum of risk exposures and disease patterns. Between June 2004 and July 2008, invitations were sent to all adult permanent, not severely disabled residents aged 35–74 y in preselected rural villages or urban residential committees. Approximately one-third (33% in rural and 27% in urban areas) responded and were enrolled in the study. In total, 512,726 participants were enrolled, including a few slightly outside the original target age range (i.e., aged 30–34 or 76–79 y). Trained health professionals administered a laptop-based questionnaire at local study assessment clinics, capturing information on sociodemographic characteristics, personal and family medical history, self-reported health status, and lifestyle factors. Standard protocols were followed for physical measurements of anthropometrics, body composition (TANITA), and blood pressure, whereas venous blood samples were collected for long-term storage and onsite blood tests, including spot test for random plasma glucose (RPG, Johnson & Johnson SureStep Plus Meter). All participants provided written informed consent, and ethical approvals were obtained at local, national, and international levels before the start of the recruitment.

### Dietary intake assessment

Dietary intake information was collected using a validated interviewer-administered laptop-based questionnaire. This included the habitual consumption frequency (5 frequency categories including daily, 4–6 d/wk, 1–3 d/wk, monthly or never/rarely) of 12 major food groups over the preceding year [21]. A validation study, conducted among 432 CKB participants during 2015–2016, demonstrated good reproducibility and validity of the questionnaire, for example, weighted kappa statistics for the total dairy consumption frequency were 0.82 and 0.75 for reproducibility and validity, respectively [22]. Furthermore, 2 resurveys were carried out in 5%–6% of randomly selected surviving participants to account for regression dilution bias by estimating long-term usual levels of various baseline exposures [23]. Additional dietary information, including daily portions of food groups and consumption of dairy subtype products, that is, cow milk, yogurt, and other dairy products (e.g., cheese and milk powder), was also collected during the second resurvey (2013–2014) among ∼25,000 participants. As previously reported [24], using the information from the second resurvey, the mean usual amount of total dairy consumption (i.e., mean intake level during the follow-up period) was estimated for each consumption frequency category at baseline.

### Follow-up for cardiometabolic morbidity and mortality

The vital status of participants was collected periodically from the Disease Surveillance Points system (local death registry) in China [25], checked annually against local residential records and health insurance data and actively confirmed by street committees or local residential administrators. Furthermore, data on hospitalized events were collected through electronic linkages via unique personal identification numbers to major chronic disease registries and the national health insurance claim system, which has almost universal (∼99%) coverage of all hospitalizations for our participants. Fatal and nonfatal events were coded according to the International Classification of Diseases, 10th revision (ICD-10) by trained clinical staff, who were blinded to baseline information [20]. The main cardiometabolic disease events examined in the present study were the incidence (both fatal and nonfatal events) of diabetes (E10-E14), IHD (ICD-10 code I20-I25), acute MI (I21), IS (I63), and ICH (I61), plus cardiovascular death (I00-I25, I27-I88, and I95-I99 as underlying cause of death). All analyses were restricted to first disease events, with cardiovascular events of IHD, acute MI, ICH, and IS being censored for each other. The disease events from enrollment until 1 January, 2019 (global censoring date) were included in these analyses, by which time only 3672 (0.8%) participants were lost to follow-up.

### Measurement of circulating biochemical markers and metabolites

Seventeen biochemical markers (Supplemental Table 1) were quantified using standard clinical biochemistry assays at the Wolfson Laboratory, CTSU, University of Oxford in baseline plasma samples of ∼18,000 CKB participants from a nested case-control study of CVD [26]. A smaller subset of CKB participants from this nested case-control study of CVD [2], [26] other different case–subcohort studies for diabetes [27] and pancreatic cancer were selected for metabolomics measurements (*n*∼7000). Nuclear magnetic resonance (NMR) spectroscopy (Nightingale Laboratory) was used to quantify simultaneously 147 metabolites and 78 derived traits, either as absolute concentrations of each metabolic measure or as ratios.

### Statistical analysis

CKB participants with major prevalent diseases, including stroke, transient ischemic attack (*n =* 8884), IHD (*n =* 15,472), cancer (*n =* 2578), or diabetes (*n =* 30,300) at baseline and those with missing values for BMI (*n =* 2) were excluded, leaving 461,046 participants in the current analysis.

Participants were classified into 4 frequency categories of dairy consumption (i.e., never/rarely, monthly, 1–3 d/wk, and ≥4 d/wk) to ensure adequate numbers of cases in each consumption category. Baseline characteristics were described as means and SDs or percentages across dairy consumption categories, with adjustments for sex, age and region as appropriate, by means of either multiple linear regression (for continuous outcomes) or logistic regression (for binary outcomes). Cross-sectional associations of dairy consumption with BMI, RPG, systolic blood pressure (SBP) and diastolic blood pressure (DBP) in each sex group were analyzed using linear regression, adjusting for age, region, education, annual household income, smoking, alcohol consumption, total physical activity, consumption of fresh fruit, red meat, poultry, fish and eggs and family history of CVD or diabetes accordingly. Analyses for RPG, SBP, and DBP were also adjusted for BMI and waist circumference (WC). In the analyses involving biochemistry and metabolomics data, all plasma biomarkers were standardized to have an SD of 1. Linear regression models were used to estimate the means of biomarker levels by dairy intake, adjusting for the aforementioned covariates plus age [2], sex, and fasting time. Inverse probability of sampling weights was applied to account for the nested case-control study design of the study on biochemical markers and metabolites, as described previously [26]. To correct for multiple testing, Benjamini–Hochberg method was used to calculate the false discovery rate (FDR) at 0.05.

Cox regression analysis was used to calculate the hazard ratios (HRs) and 95% confidence intervals (CIs) for dairy consumption in relation to each cardiometabolic disease outcome, stratified by age-at-risk (in 5-y intervals), sex, and region and adjusted for the aforementioned potential confounders plus baseline age and BMI. Blood pressure (SBP) was also added in as a covariate (Supplemental Table 2). The floating absolute risk method, which provides variance of log risk for each category (including the reference group), was used to facilitate comparisons between any 2 exposure groups rather than just with an arbitrarily chosen reference group [28]. The dairy consumption was entered into models as either a categorical variable or as a continuous variable, which was used to estimate the HRs for each 50 g/d increment in usual dairy consumption to quantify the linear association and to account for regression dilution bias [23].

Subgroup analyses by baseline factors (e.g., age-at-risk, sex, and region) were performed, and Chi-square values for trend or heterogeneity were calculated. The proportional hazards assumption was tested by comparing the HRs for the first and second halves of follow-up, with no strong evidence of departure being observed. Sensitivity analyses were done by additionally adjusting for other covariates, including other dietary variables, anthropometric factors, and RPG and by excluding the first 2 y of follow-up. All analyses were carried out using SAS (version 9.4, SAS Institute), and figures were generated using R 4.3.3 (https://www.R-project.org/).

## Results

Among the 461,046 participants included in the study, the mean baseline age was 51.2 (SD 10.5), and the estimated usual mean dairy consumption was 41.7 g/d (Table 1). At baseline, 10.7% of the participants were regular consumers (i.e., those who reported consuming dairy ≥4 d/wk), whereas 70.0% were nonconsumers (i.e., reported never or rare consumption). Participants with more frequent dairy intake were more likely to be female, urban residents, had higher education and annual household income levels, and included a higher proportion of regular consumers of red meat, fish, and fresh fruit. The mean values of total physical activity (metabolic equivalent-h/d), WC, and RPG were similar across the 4 dairy intake groups.

**TABLE 1:** Characteristics of participants by frequency of dairy intake at baseline survey (2004–2008)

Characteristic	Frequency of dairy intake				Overall (*n* = 461,046)
	Never/rarely	Monthly	1–3 d/wk	≥4 d/wk	
	(*n* = 322,781)	(*n* = 50,754)	(*n* = 38,265)	(*n* = 49,246)	
Usual dairy intake^1^ (g/d)	24.9	47.1	79.5	116.7	41.7
Mean age (SD) (y)	51.2 (10.8)	50.8 (10.6)	49.8 (10.9)	52.5 (11.2)	51.2 (10.5)
F**emales** (%)	58.3	57.3	60.2	64.1	59.0
Urban (%)	29.7	53.3	81.6	82.9	42.3
Education >6 y (%)	42.6	55.6	68.5	72.8	49.4
Household income >20,000 yuan/y (%)	36.0	51.7	58.7	64.2	42.6
Ever regular smoking in males^2^ (%)	76.2	73.7	68.2	69.0	74.6
Ever regular alcohol drinking in males^3^ (%)	38.7	34.7	32.7	33.4	37.2
Family history of CVD (%)	19.4	20.3	20.7	22.5	19.9
Regular dietary intake (%)^4^					
Red meat	44.7	48.6	51.5	56.4	47.0
Fish	7.8	9.3	10.3	14.7	8.9
Fresh fruit	21.5	28.3	41.6	56.5	27.7
Mean physical activity (SD), MET-h/d	22.1 (12.6)	21.6 (12.3)	21.0 (12.6)	21.2 (13.0)	21.9 (13.9)
Mean BMI (SD) (kg/m^2^)	23.6 (3.3)	23.5 (3.3)	23.3 (3.4)	23.2 (3.5)	23.5 (3.3)
**Mean WC (SD) (cm)**	79.9 (9.5)	79.9 (9.3)	79.5 (9.6)	79.0 (9.9)	79.7 (9.6)
Mean RPG (SD) (mmol/L)^5^	5.7 (1.2)	5.7 (1.1)	5.7 (1.2)	5.6 (1.2)	5.7 (1.1)
Mean SBP (SD) (mmHg)	130.9 (20.1)	129.2 (19.6)	128.0 (20.2)	126.8 (20.8)	130.0 (20.8)
Mean DBP (SD) (mmHg)	77.9 (11.3)	77.1 (11.1)	76.6 (11.4)	76.1 (11.7)	77.5 (11.1)
Self-rated poor health (%)	9.0	8.8	7.7	8.2	8.8

Linear regression (for continuous outcomes) or logistic regression (for binary outcomes) were used to calculate the means (SDs) or percentages of various baseline characteristics across 4 frequency categories of dairy consumption (i.e., never/rarely, monthly, 1–3 d/wk and ≥4 d/wk), with adjustments for age (continuous), sex (dichotomous variable) and region (10 regions), where appropriate.

Abbreviations: CVD, cardiovascular disease; DBP, diastolic blood pressure; MET, metabolic equivalent of task; RPG, random plasma glucose; SBP, systolic blood pressure; WC, waist circumference.

1 Crude mean values from second resurvey (2013–2014) of randomly selected 20,084 participants without CVD, diabetes, or cancer at either baseline or second resurvey.

2 Percentage values of ever regular smoking in females were 3.2%, 2.9%, 2.2%, 2.0%, and 3.0% for dairy intake of “never/rarely,” “monthly,” “1–3 d/wk,” “≥4 d/wk” and overall, respectively.

3 Percentage values of ever regular alcohol drinking in females were 2.3%, 2.4%, 2.7%, 3.6%, and 2.5% for dairy intake of “never/rarely,” “monthly,” “1–3 d/wk,” “≥4 d/wk” and overall, respectively.

4 Percentage values of participants who reported ≥4 d/wk intake.

5 Values for RPG were missing for 7961 participants.

Mean levels of dairy intake varied largely across the 10 regions in both sexes, with the proportion of regular dairy consumers being 45-fold different in males and 32-fold different in females, and with Qingdao having the highest (followed by Harbin) and Zhejiang having the lowest (followed by Hunan) proportions (Supplemental Figure 1). Between rural and urban areas, the proportion of dairy consumers also showed distinct age distribution patterns. In urban areas, there was a slightly higher proportion of females than males before the age of ∼60 y, but this sex difference disappeared after ∼60 y. However, in rural areas, there was a gradual increase in the proportion of regular dairy consumers with age in both males and females (Supplemental Figure 2). Among the 5 urban regions, Suzhou and Haikou (where a large proportion of participants came from suburban areas) had the lowest values of estimated mean dairy intake in both sexes (Supplemental Figure 3). Among 11,692 participants who answered 3 questionnaire surveys, there was an increasing trend of dairy consumption (Supplemental Figure 4) and milk was the most frequently consumed dairy product, followed by yogurt (Supplemental Figure 5).

After adjustment for potential confounders, dairy consumption was significantly and inversely associated with BMI, SBP, and DBP in both sexes, with regular consumers having a 0.5 and 0.6 kg/m^2^ lower BMI, 2.2 and 1.9 mmHg lower SBP, and 1.4 and 1.0 mmHg lower DBP than nonconsumers in males and females, respectively. No clear association was evident between dairy consumption and RPG (Supplemental Figure 6).

**FIGURE 1:**
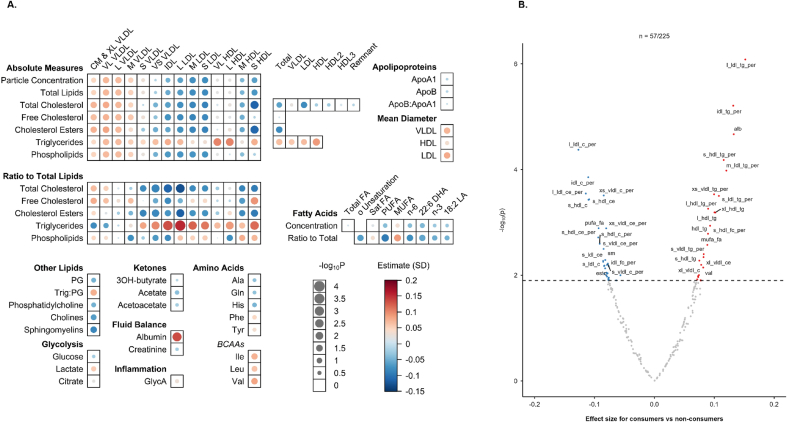
Associations of dairy intake with 225 NMR circulating metabolites and derived traits at baseline survey (2004–2008). (A) Bubble heatmap showing the associations of dairy intake [consumers (*n =* 1510) compared with nonconsumers (*n =* 4543)] with metabolomic markers. Negative log10 *P* values represent the FDR-corrected *P* values, and the size of the bubble is inversely proportional to the *P* value, indicating the strength of association (smaller *P* values correspond to larger bubbles). The color gradient represents the magnitude and direction of the associations, with red indicating positive associations and blue indicating negative associations. The intensity of the color corresponds to the magnitude of the effect: darker shades signify stronger associations, whereas lighter shades approach neutrality. White represents a neutral or zero effect. (B) Volcano plot showing the top dairy-associated metabolites. The *x*-axis represents the effect size of the association between dairy (consumers vs. nonconsumers) and the metabolites, whereas the *y*-axis indicates the negative log10 *P* values (FDR-corrected values). Red dots denote positive FDR-corrected associations, blue dots denote negative FDR-corrected associations, and grey dots denote nonsignificant associations. Values of circulating metabolites were standardized and analysis was adjusted for age (continuous), age^2^ (continuous), sex (dichotomous), region (10 regions), fasting time (continuous), batch number (3 categories), education (4 categories), income (4 categories), smoking (4 categories), alcohol consumption (4 categories), total physical activity (continuous), family history of CVD (dichotomous) and consumption of fresh fruit (5 categories), red meat (4 categories), poultry (3 categories), fish (4 categories) and eggs (4 categories) and BMI (continuous). Inverse probability of sampling weighting was used to account for the ascertainment status of the participants. Participants with prevalent CVD, diabetes, or cancer were excluded from the analysis. APOA1, apolipoprotein A-I; APOB apolipoprotein B; APOB:APOA1, ratio of apolipoprotein B to apolipoprotein A-I; CVD, cardiovascular disease; FDR, false-discovery rate; IDL, intermediate-density lipoprotein; L, large; M, medium; S, small; VL, very large; VS, very small; XL, extra large.

Dairy intake was positively associated with levels of total triglycerides, apolipoprotein A1, albumin (ALB), and uric acid (Supplemental Table 1 and Supplemental Figure 7). The majority of these associations tended to be linear, with regular dairy consumers having higher levels of these biochemical markers than nonconsumers (Supplemental Figure 7). Among the 225 NMR-based circulating metabolites and derived traits, dairy intake was significantly associated with levels of 57 metabolites after FDR correction (Figure 1A and B). In particular, dairy consumption was positively associated with total triglycerides, triglycerides in all lipid measures of all 14 lipoprotein subclasses, ALB, and valine, but inversely associated with sphingomyelins, absolute measures and ratios of total cholesterol and cholesterol esters in small and very small VLDL, intermediate-density lipoprotein, small LDL and small HDL as well as PUFAs to total fatty acids ratio.

Over a mean follow-up period of 11.8 (SD 2.1) y, there were 18,306 diabetes cases, 33,946 IHD (including 3888 acute MI), 33,670 IS, 7191 ICH, and 13,241 cardiovascular deaths recorded. Before adjusting for BMI, dairy consumption was positively associated with IHD but inversely associated with all other cardiometabolic disease endpoints under investigation (Table 2). Compared with nonconsumers, regular dairy consumers had 9% (95% CI: 6%, 12%) higher risk of IHD but 11% (6%, 15%), 11% (2%, 20%), 6% (3%, 9%), 32% (25%, 39%) and 19% (13%, 24%) lower risk of diabetes, acute MI, IS, ICH, and cardiovascular death, respectively. Additional adjustment for BMI greatly attenuated the association with diabetes to null, but did not essentially modify the associations with other CVD outcomes, although the association between dairy intake and IS became only borderline significant [0.96 (0.93, 0.99) *P*_trend_ = 0.13]. When BMI and other potential confounders were taken into account, each 50 g/d higher usual dairy intake was associated with 6% (4%, 8%) higher risk of IHD, but 7% (1%, 12%) lower risk of acute MI, 2% (0%, 4%) lower risk of IS, 17% (12%, 21%) lower risk of ICH, and 10% (6%, 13%) lower risk of cardiovascular death. Furthermore, adjustment for SBP only essentially altered the associations of dairy consumption with ICH (from 17% to 12%) and cardiovascular death (from 10% to 6%), but not others (Supplemental Table 2).

**TABLE 2:** Adjusted HRs of major cardiometabolic diseases^1^ associated with dairy intake^2^

Cardiometabolic disease type	Dairy products intake				*P*-trend	HR (95% CI) per 50 g/d of usual dairy intake
	Never/rarely	Monthly	1–3 d/wk	≥4 d/wk		
Diabetes						
No. of events	12,855	1744	1347	1718		
Model 1^3^	1.00 (0.97, 1.03)	0.99 (0.95, 1.04)	0.97 (0.92, 1.02)	0.89 (0.85, 0.94)	0.0005	0.95 (0.92, 0.98)
Model 2^4^	1.00 (0.97, 1.03)	1.04 (0.99, 1.09)	1.04 (0.98, 1.10)	1.00 (0.95, 1.06)	0.50	1.01 (0.98, 1.04)
Heart disease						
IHD						
No. of events	20,781	4031	3485	5649		
Model 1^3^	1.00 (0.98–1.02)	1.05 (1.02, 1.08)	1.08 (1.04, 1.11)	1.09 (1.06, 1.12)	<0.0001	1.05 (1.03, 1.07)
Model 2^4^	1.00 (0.98, 1.02)	1.05 (1.02, 1.09)	1.09 (1.05, 1.13)	1.11 (1.08, 1.15)	<0.0001	1.06 (1.04, 1.08)
Acute MI						
No. of events	2699	472	271	446		
Model 1^3^	1.00 (0.95, 1.05)	1.03 (0.94, 1.13)	0.93 (0.82, 1.05)	0.89 (0.80, 0.98)	0.05	0.93 (0.88, 0.99)
Model 2^4^	1.00 (0.95, 1.05)	1.03 (0.94, 1.13)	0.92 (0.81, 1.04)	0.88 (0.80, 0.98)	0.04	0.93 (0.88, 0.99)
Stroke						
IS						
No. of events	22,332	4208	2883	4247		
Model 1^3^	1.00 (0.98, 1.02)	1.04 (1.01, 1.07)	0.99 (0.95, 1.03)	0.94 (0.91, 0.97)	0.006	0.97 (0.95, 0.99)
Model 2^4^	1.00 (0.98, 1.02)	1.05 (1.01, 1.08)	1.00 (0.96, 1.04)	0.96 (0.93, 0.99)	0.13	0.98 (0.96, 1.00)
ICH						
No. of events	5693	724	357	417		
Model 1^3^	1.00 (0.96, 1.04)	0.92 (0.85, 0.98)	0.85 (0.76, 0.95)	0.68 (0.61, 0.75)	<0.0001	0.83 (0.78, 0.87)
Model 2^4^	1.00 (0.96, 1.04)	0.92 (0.86, 0.99)	0.86 (0.77, 0.95)	0.69 (0.62, 0.76)	<0.0001	0.83 (0.79, 0.88)
Cardiovascular death						
No. of events	10,030	1465	693	1026		
Model 1^3^	1.00 (0.97, 1.03)	0.99 (0.94, 1.04)	0.88 (0.82, 0.95)	0.81 (0.76, 0.87)	<0.0001	0.90 (0.87, 0.93)
Model 2^4^	1.00 (0.97, 1.03)	0.99 (0.94, 1.04)	0.89 (0.82, 0.96)	0.82 (0.77, 0.87)	<0.0001	0.90 (0.87, 0.94)

Abbreviations: CI, confidence interval; CVD, cardiovascular disease; IHD, ischemic heart disease; HR, hazard ratio; ICH, intracerebral hemorrhage; IS, ischemic stroke; MI, myocardial infarction.

1 Events of IHD, acute MI, ICH, and IS were censored for each other.

2 Analysis was performed among 461,047 participants with no prior self-reported history of CVD, diabetes or cancer at baseline.

3 Model 1: analysis was stratified by age-at-risk (continuous variable), sex (dichotomous variable) and individual regions (10 regions) and were adjusted for baseline age (continuous), education (4 categories), income (4 categories), smoking (4 categories), alcohol consumption (4 categories), total physical activity (continuous variable), family history of CVD (dichotomous variable), consumption of fresh fruit (5 categories), red meat (4 categories), poultry (3 categories), fish (4 categories), and eggs (4 categories).

4 Model 2: as for model 1, additionally adjusted for BMI (continuous variable).

**FIGURE 2:**
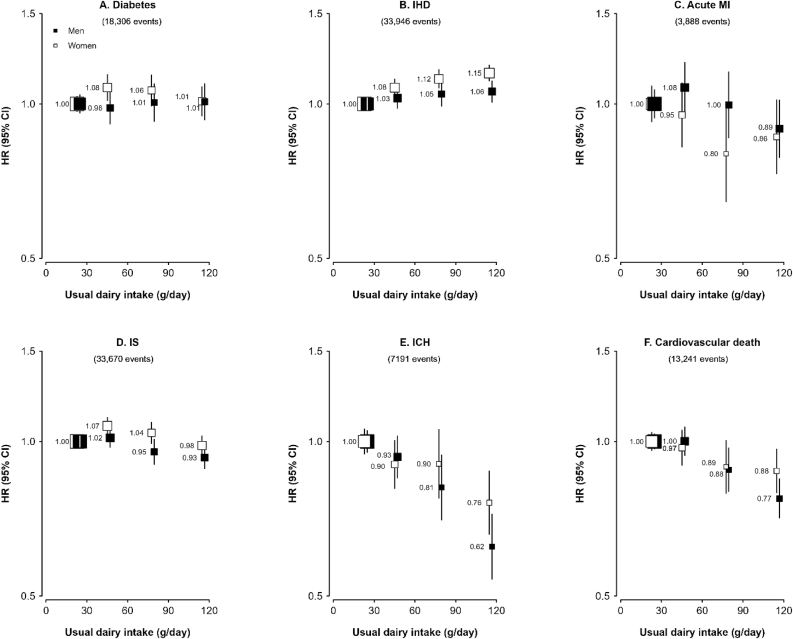
Associations of usual dairy intake (g/d) with incidence of (A) diabetes, (B) IHD, (C) acute MI, (D) IS, (E) ICH, and (F) cardiovascular death, by sex. Events of IHD, acute MI, IS, and ICH were censored for each other. Analysis was stratified by age-at-risk (continuous), sex (dichotomous), and region (10 regions) and were adjusted for baseline age (continuous), education (4 categories), income (4 categories), smoking (4 categories), alcohol consumption (4 categories), total physical activity (continuous), family history of CVD or diabetes (dichotomous), consumption of fresh fruit (5 categories), red meat (4 categories), poultry (3 categories), fish (4 categories) and eggs (4 categories) and BMI (continuous). The *y*-axis was plotted on a log scale with the lowest intake group as a reference category. The squares represent HRs with the size being inversely proportional to the variance of the log of HR, and the vertical lines represent 95% CIs. The numbers next to the squares are point estimates for HRs. Solid squares represent males and open squares represent females. CI, confidence interval; CM, chylomicrons; CVD, cardiovascular disease; HR, hazard ratio; ICH, intracerebral hemorrhage; IHD, ischemic heart disease; IS, ischemic stroke; MI, myocardial infarction.

**FIGURE 3:**
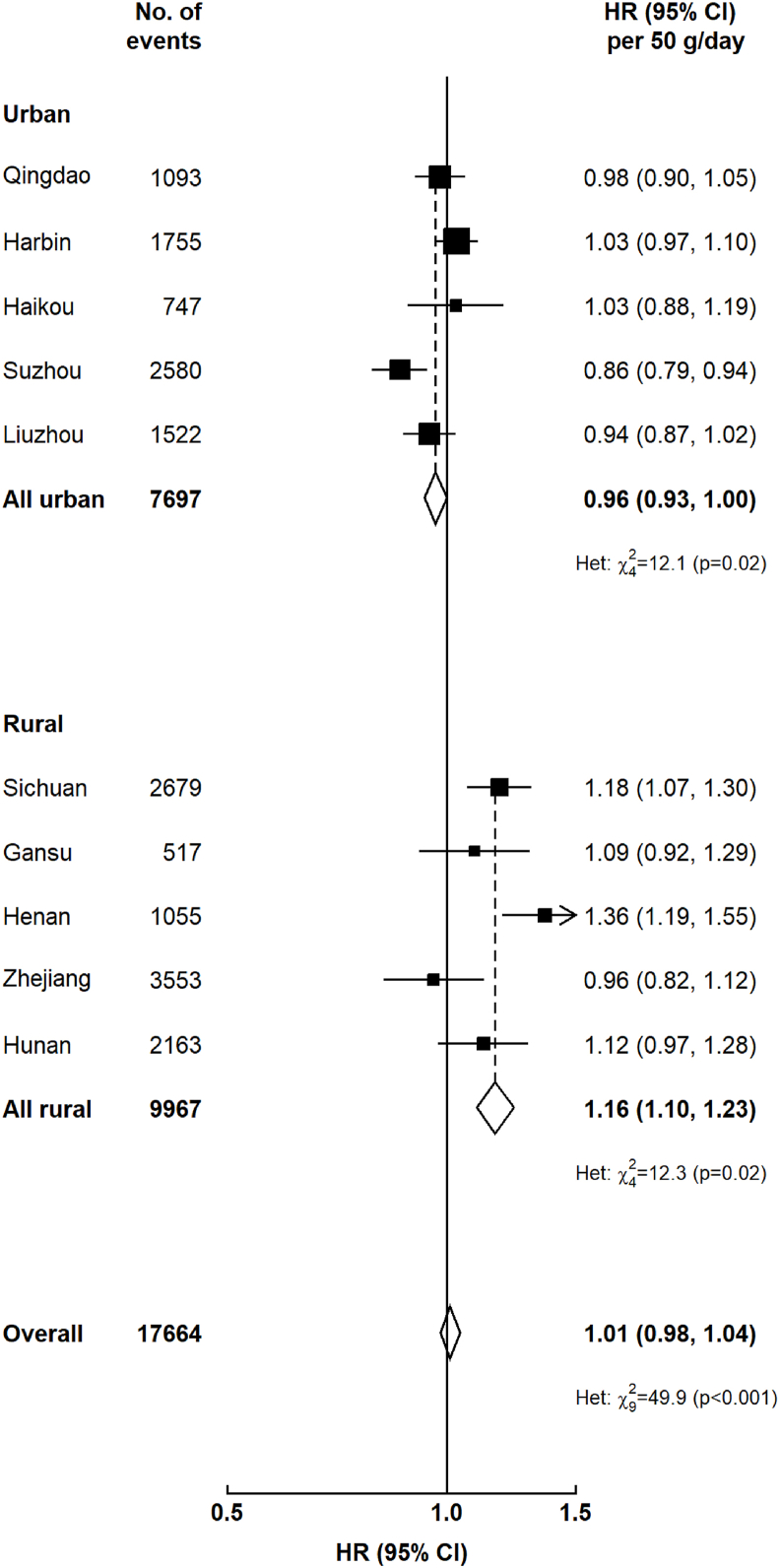
Adjusted HRs (95% CIs) for diabetes per 50 g/d of usual dairy intake by region. Analysis was stratified by age-at-risk (continuous), sex (dichotomous), and region (10 regions) and were adjusted for baseline age (continuous), education (4 categories), income (4 categories), smoking (4 categories), alcohol consumption (4 categories), total physical activity (continuous), family history of diabetes (dichotomous), consumption of fresh fruit (5 categories), red meat (4 categories), poultry (3 categories), fish (4 categories) and eggs (4 categories) and BMI (continuous). Overall HR per 50 g/d usual dairy intake after correcting for regression dilution bias. Black squares represent HRs (size is inversely proportional to the variance of the log of HR); horizontal lines represent 95% CIs; white diamonds represent overall HRs (95% CIs); “No of events” refers to the number of incident diabetes events in each group; the subscript numbers in the Chi-square values represent the degrees of freedom. CI, confidence interval; Het, heterogeneity; HR, hazard ratio.

The positive association between consumption of dairy products and risk of IHD was more pronounced in females than in males (HR = 1.15 in females compared with 1.06 in males for regular compared with nonconsumers, *P*_heterogeneity_ = 0.03), but no such sex difference was observed in the associations of dairy consumption with other disease outcomes (Figure 2). In addition, stratified analyses by study area showed dairy consumption to be inversely associated with diabetes risk in urban areas, but positively associated in rural areas (Supplemental Figure 8). No such pronounced differences were found in associations of dairy consumption with other CVD endpoints (Supplemental Figure 8 and Supplemental Figure 9). Furthermore, region-specific analysis on diabetes found that the inverse association between dairy consumption and diabetes risk in urban areas was mainly driven by data from Suzhou [HR per 50 g/d dairy intake = 0.86 (0.79, 0.94)], whereas the positive association in rural areas was mainly driven by data from Sichuan and Henan, with HRs being 1.18 (1.07, 1.30) and 1.36 (1.19, 1.55), respectively, for each 50 g/d increase in usual dairy intake (Figure 3).

The associations of dairy intake with risks of acute MI, ICH, and cardiovascular death tended to be inverse in almost all of the subgroups by baseline factors, with no statistically significant heterogeneities (Bonferroni-corrected *P* <0.001 = 0.05/63 subgroup analyses) being observed (Supplemental Figures 10–12). However, the positive association of dairy intake with IHD risk was more pronounced in never regular alcohol drinkers and people with lower annual household income status (*P*_heterogeneity or trend_ <0.001, respectively; Supplemental Figure 13). The observed associations remained essentially unchanged across various sensitivity analyses with further exclusions and adjustments (Supplemental Table 3).

## Discussion

In this comprehensive study involving a large number of Chinese adults with relatively low dairy intake, higher dairy consumption was associated with higher risk of IHD but with lower risks of diabetes, acute MI, IS, ICH, and cardiovascular death, with regular consumption (compared with no consumption) being associated with HRs of 1.09, 0.89, 0.89, 0.94, 0.68, and 0.81, respectively, before adjusting for BMI. However, after additional adjustment for BMI, the overall association with diabetes was attenuated to null, whereas the associations with CVD outcomes remained largely unchanged. Besides an inverse association with BMI, dairy intake was also inversely associated with SBP and DBP. Nonetheless, the prospective associations observed were largely independent of SBP, suggesting the involvement of other mechanisms through which dairy consumption could influence CVD risks. The blood biomarkers including ALB, triglycerides, small LDL particles, and total cholesterol and cholesterol esters in small HDL might also play a role in the mechanisms underlying the association between dairy consumption and CVD risk.

Consistent with previous studies in China [18,29,30], we observed noteworthy differences in patterns of dairy intake by sex, age, and rural/urban residence, with much higher intake among urban areas than in rural areas. In addition, as reported previously [31], dairy intake in China [18] has been rising in the past decades, although it still remains much lower than in the West [16,32]. All these likely suggest that socioeconomic status and availability of dairy products [13,29,33,34] might be the main determinants of dairy consumption, rather than lactase persistence [35].

Our findings of the inverse association between dairy consumption and BMI, as well as blood pressure, align with previous research in Chinese populations [[36], [37], [38]] and previous meta-analyses of cohort studies [6,39]. The inverse association with BMI might be partly due to the protein content in dairy products that can enhance satiety and reduce overall caloric intake [40]. Moreover, the potential impacts of dairy products on blood pressure regulation might be partially attributed to minerals, such as calcium and potassium, and bioactive peptides in dairy products, which have been shown to have antihypertensive properties [41,42]. Mendelian randomization (MR) studies, however, showed a positive association between genetically predicted milk intake and BMI [43,44], and a nonsignificant association between milk intake and blood pressure [45], with the majority of these studies being conducted in Western populations. Among the Chinese population and in CKB, >99% of the individuals have the lactase nonpersistence genotype [15,35], limiting therefore the possibility of conducting MR analysis to confirm/refute the causal role of dairy intake in health, for example, body weight and blood pressure management.

The null association between dairy consumption and diabetes after adjustment for BMI in our study is somewhat in line with the evidence from a prospective study in China, which included 22,843 participants (735 incident diabetes cases), where the associations of total dairy, milk, and yogurt with diabetes incidence were attenuated by additionally adjusting for BMI [46]. This finding and the results from previous prospective studies conducted in Europe [47,48] suggest a strong confounding and/or mediating role of BMI in diet–diabetes association, and more particularly in the association with dairy intake. A recent systematic review of 6 MR studies in Western populations also revealed a null association between milk intake and diabetes risk, highlighting the potential confounding effect that BMI and lipid metabolism have on the genetic variant–diabetes association [49]. However, we cannot clearly explain the significant, but in opposite direction, associations between dairy consumption and diabetes risk after adjusting for BMI in 3 of 10 CKB study areas. This could be a chance finding or due to some unknown factors, for example, gut microbiota variations. For instance, a previous study in China showed that the residential area of participants had the strongest impact on associations between gut microbiome composition and metabolic diseases [50]. In addition, the observed geographic heterogeneity may partly reflect regional differences in the types of dairy products consumed, which could not be examined in the present study. Future gut microbiome studies and more detailed intake assessment of various types of dairy products in CKB would provide more insights into the distinct association between dairy intake and diabetes across different regions.

The observed inverse association between dairy intake and risk of acute MI in the current study is in line with rather limited previous evidence from a Swedish cohort study [51], although the dairy intake examined in this study was specified to be fermented dairy intake. Nonetheless, a recent meta-analysis of 6 prospective cohort studies from the United States, Europe, and Iran (16,478 cases) reported that higher consumption of high-fat milk was associated with a higher risk of coronary artery disease (CAD) [11], which is consistent with the positive association between dairy intake and IHD risk observed in our study. However, the same meta-analysis and another recent meta-analysis have also reported moderate inverse associations of cheese intake (7 cohort studies with 14,698 cases) [11] and of total dairy consumption (24 risk estimates from cohort studies in United States, Europe, and Asia with 34,248 cases) [6] with risk of CAD, respectively. Furthermore, another recent prospective study in China observed no association with milk intake and IHD risk [52]. Therefore, there is still no consistent global evidence on the potential role of dairy consumption in IHD development. The observed effect modifications by sex and annual household income in our study, as well as the opposite associations of dairy consumption with acute MI and IHD, suggest that the inconsistent associations reported in the literature might stem from residual confounding by sociodemographic factors and from differences in IHD outcome definition.

Fewer number of studies have investigated the association between dairy intake and stroke subtypes (ischemic and ICH). A large prospective study of 9 European countries [8] and a meta-analysis (combining the results from 3 cohort studies from Europe and North America with 3691 cases) [11] have reported a moderate inverse association between milk intake and IS risk, that is, 4%–7% lower risk per 200 g/d higher milk intake, whereas others have found no significant association [7,53]. Although the inverse association observed in our study was attenuated to be only borderline significant after adjusting for BMI, the strength of association (i.e., 2% lower risk per 50 g/d intake) was similar to those of the previous studies. With respect to ICH risk, we found a much stronger inverse association with dairy consumption, compared with the findings of another recent Chinese study (664 hemorrhagic stroke cases), which showed that there was a 14% lower risk per 100 g/d higher milk intake [52]. Furthermore, vegans (not consuming any animal foods including dairy) in EPIC-Oxford study had a higher risk of hemorrhagic stroke than meat eaters (consuming all animal foods), which somewhat aligns with our results, although the estimates were not statistically significant due to the very small number of cases (173 and 8 hemorrhagic stroke cases in meat eaters and vegans, respectively) [54].

The inverse association of dairy consumption with cardiovascular death (30.6% due to ICH) is consistent with, but much stronger than the 7% lower risk of CVD mortality for highest compared with lowest total dairy consumption, which was previously reported in a meta-analysis of 16 cohort studies (29,359 CVD mortality cases) [55]. Two prospective studies with Chinese adults also reported an inverse association of milk intake with CVD mortality risk [52,56].

Several mechanisms may underlie the mostly inverse associations between dairy intake and CVD subtypes observed in the current study, which were largely independent of adiposity and blood pressure. First, the higher ALB levels in dairy consumers compared with nonconsumers may explain at least partially the observed inverse associations. Previous studies have found that plasma ALB levels were inversely associated with the incidence of various CVD types [57] and MR studies demonstrated that genetically determined low serum ALB concentration was associated with increased risks of certain CVDs, including stroke [58] and hypertension [59]. Second, dairy consumption was associated with lower plasma levels of total cholesterol and cholesterol esters in small HDL. Previous studies have shown that smaller HDL particles are associated with a higher CVD risk, whereas larger HDL particles are associated with a lower CVD risk [60]. Third, dairy intake was inversely associated with concentration of total cholesterol and cholesterol esters in small and very small VLDL, which have been reported to be markers of residual atherosclerotic CVD risk [61]. A reduction in small VLDL could be associated with a reduction in residual atherosclerotic CVD risk, independent of changes in LDL cholesterol levels [61]. Lastly, additional mechanisms potentially underlying the observed inverse dairy-CVD associations might involve vitamin B12 [62], essential amino acids [63], calcium, potassium, and bioactive peptides [41] contained in dairy products and the beneficial alterations in the gut microbiome associated with higher milk intake [64]. However, the evidence gap is still large and further studies (including MR studies to establish causality of associations) are needed to better understand these associations. Such knowledge would also help us to interpret the conflicting findings in dairy consumption with blood triglycerides (positive) and CVD risk (largely inverse) in the current study, as available evidence generally supports that blood levels of triglycerides are a risk factor for CVD conditions [65].

Strengths of this study include its large sample size, prospective design, long follow-up period, comprehensive adjustment for a wide range of potential confounders and investigating the potential impact of reverse causality. The use of repeated measurements for the dairy intake also allowed us to account for regression dilution bias. Additionally, our study population had relatively low dairy consumption compared with Western populations, which could enhance the generalizability of our findings to other populations with low dairy intake, for example, some vegetarians such as ovo-vegetarians and East Asian populations. However, several limitations should be considered. First, the study's dietary data at baseline were based only on a rather crude frequency questionnaire collecting information on consumption of major food groups rather than individual food items, limiting the ability to adjust for specific nutrients and total energy intake. Second, the associations between intake of different types of dairy products (i.e., milk, yogurt, and other dairy products) and cardiometabolic disease risk could not be reliably assessed, because this information was only available among a subset of CKB participants who attended the second resurvey in 2013–2014. Therefore, the sample size is relatively small (*n* ∼20,000), and the follow-up duration is short (i.e., mean ∼4 y of follow-up). Third, the observed cross-sectional association between dairy consumption and biomarkers (particularly NMR metabolites) was of very weak strength. Such findings need to be interpreted with caution as the differences in dairy consumers and nonconsumers may also reflect the dairy-independent differences between lactase persistent and lactase nonpersistent individuals [15]. Lastly, residual confounding cannot be ruled out, despite adjusting for a wide range of covariates in our analysis, and thus causality of associations could not be robustly established.

In conclusion, our study in a large sample of Chinese adults with relatively low dairy (mainly milk and yogurt) intake showed that a higher consumption of dairy products was associated with a higher risk of IHD but lower risks of acute MI, ICH, and cardiovascular death independent of adiposity and blood pressure. Future studies are warranted to further elucidate these relationships and their causality, investigate the impact of specific dairy products (e.g., milk, cheese, and yogurt) on various cardiometabolic outcomes and to explore the underlying mechanisms in greater detail. These findings, in combination with future research, might inform dietary recommendations aimed at improving cardiovascular health in China, where the levels of dairy intake are low but increasing.

## Author contributions

The authors’ responsibilities were as follows – MGK: analyzed the data, prepared the figures/tables and drafted the manuscript; MGK, HD: contributed to the conception of this paper, interpretation of the results and the revision of manuscript; HD, HW, AI, YC, CK, PKI, IYM, KP, CY, PP, DS, LY, MY, JL: were involved in data collection/management and development of analytical method; XY, DA: provided administrative and technical support; ZC, LL, JC: designed the CKB study and were involved in funding and data acquisition; and all authors: critically reviewed the manuscript and approved the final submission.

## Data availability

The CKB is a global resource for the investigation of lifestyle, environmental, blood biochemical and genetic factors as determinants of common diseases. The CKB study group is committed to making the cohort data available to the scientific community in China, the UK and worldwide to advance knowledge about the causes, prevention and treatment of disease. For detailed information on what data are currently available to open access users and how to apply for it, visit: https://www.ckbiobank.org/data-access. Researchers who are interested in obtaining the raw data from the CKB study that underlines this paper should contact ckbaccess@ndph.ox.ac.uk. A research proposal will be requested to ensure that any analysis is performed by bona fide researchers and—where data are not currently available to open access researchers—is restricted to the topic covered in this paper.

## Ethics approval and consent to participate

All participants provided informed consent. Ethics approvals were obtained at a local, national, and international level before the beginning of recruitment. Ethics approval was particularly obtained from the Ethical Review Committee of the Chinese Centre for Disease Control and Prevention (Beijing, China, 005/2004), and the Oxford Tropical Research Ethics Committee, University of Oxford (UK, 025-04).

## Consent for publication

Not applicable.

## Open access statement

This research was funded in whole, or in part, by Wellcome grants to University of Oxford (Our Planet Our Health Livestock, Environment and People—LEAP, 205212/Z/16/Z and 212946/Z/18/Z). For the purpose of open access, the author has applied a CC-BY public copyright license to any Author Accepted Manuscript version arising from this submission.

## Funding

The CKB baseline survey and the first resurvey were supported by the Kadoorie Charitable Foundation in Hong Kong. The long-term follow-up has been supported by Wellcome grants to University of Oxford (212946/Z/18/Z, 202922/Z/16/Z, 104085/Z/14/Z, 088158/Z/09/Z) and grants from the National Natural Science Foundation of China (82192900, 82192901, 82192904, 82388102) and the Noncommunicable Chronic Diseases-National Science and Technology Major Project (2023ZD0510100). The UK Medical Research Council (MC_UU_00017/1, MC_UU_12026/2, MC_U137686851), Cancer Research UK (C16077/A29186; C500/A16896), and the British Heart Foundation (CH/1996001/9454), provide core funding to the Clinical Trial Service Unit and Epidemiological Studies Unit at the University of Oxford for the project. This research was also supported by the Wellcome Trust, Our Planet Our Health (Livestock, Environment and People - LEAP) (205212/Z/16/Z). PKI is funded by a Wellcome Career Development Award (302990/Z/23/Z). The funding organizations had no role in study design; data collection, analysis and interpretation; writing of the manuscript; and decision to submit the manuscript for publication. The first and corresponding authors had full access to the data and responsibility for the final decision to submit for publication.

## Conflict of interest

The authors report no conflicts of interest.

## References

[1] Roth G.A., Abate D., Abate K.H., Abay S.M., Abbafati C., Abbasi N. (2018). Global, regional, and national age-sex-specific mortality for 282 causes of death in 195 countries and territories, 1980–2017: a systematic analysis for the Global Burden of Disease Study 2017. Lancet.

[2] Yang G., Wang Y., Zeng Y., Gao G.F., Liang X., Zhou M. (2013). Rapid health transition in China, 1990-2010: findings from the Global Burden of Disease Study 2010. Lancet.

[3] Mozaffarian D. (2016). Dietary and policy priorities for cardiovascular disease, diabetes, and obesity. Circulation.

[4] Feng Y., Zhao Y., Liu J., Huang Z., Yang X., Qin P. (2022). Consumption of dairy products and the risk of overweight or obesity, hypertension, and type 2 diabetes mellitus: a dose-response meta-analysis and systematic review of cohort studies. Adv. Nutr..

[5] Zhang X., Chen X., Xu Y., Yang J., Du L., Li K. (2021). Milk consumption and multiple health outcomes: umbrella review of systematic reviews and meta-analyses in humans. Nutr. Metab..

[6] Chen Z., Ahmed M., Ha V., Jefferson K., Malik V., Ribeiro P.A.B. (2022). Dairy product consumption and cardiovascular health: a systematic review and meta-analysis of prospective cohort studies. Adv. Nutr..

[7] de Goede J., Soedamah-Muthu S.S., Pan A., Gijsbers L., Geleijnse J.M. (2016). Dairy consumption and risk of stroke: a systematic review and updated dose-response meta-analysis of prospective cohort studies. J. Am. Heart Assoc..

[8] Tong T.Y.N., Appleby P.N., Key T.J., Dahm C.C., Overvad K., Olsen A. (2020). The associations of major foods and fibre with risks of ischaemic and haemorrhagic stroke: a prospective study of 418 329 participants in the EPIC cohort across nine European countries. Eur. Heart J..

[9] Guo J., Astrup A., Lovegrove J.A., Gijsbers L., Givens D.I., Soedamah-Muthu S.S. (2017). Milk and dairy consumption and risk of cardiovascular diseases and all-cause mortality: dose-response meta-analysis of prospective cohort studies. Eur. J. Epidemiol..

[10] Soedamah-Muthu S.S., de Goede J. (2018). Dairy consumption and cardiometabolic diseases: systematic review and updated meta-analyses of prospective cohort studies. Curr. Nutr. Rep..

[11] Jakobsen M.U., Trolle E., Outzen M., Mejborn H., Grønberg M.G., Bøge Lyndgaard C. (2021). Intake of dairy products and associations with major atherosclerotic cardiovascular diseases: a systematic review and meta-analysis of cohort studies. Sci. Rep..

[12] Thorning T.K., Bertram H.C., Bonjour J.-P., de Groot L., Dupont D., Feeney E. (2017). Whole dairy matrix or single nutrients in assessment of health effects: current evidence and knowledge gaps. Am. J. Clin. Nutr..

[13] He Y., Yang X., Xia J., Zhao L., Yang Y. (2016). Consumption of meat and dairy products in China: a review. Proc. Nutr. Soc..

[14] Huang L., Wang Z., Wang H., Zhao L., Jiang H., Zhang B. (2021). Nutrition transition and related health challenges over decades in China. Eur. J. Clin. Nutr..

[15] Misselwitz B., Butter M., Verbeke K., Fox M.R. (2019). Update on lactose malabsorption and intolerance: pathogenesis, diagnosis and clinical management. Gut.

[16] FAO. “Per capita consumption of milk, excluding butter – FAO” (2024). https://ourworldindata.org/grapher/per-capita-milk-consumption.

[17] CCDC (2024). https://en.chinacdc.cn/health_topics/nutrition_health/202206/t20220616_259702.html.

[18] Yang S., Bhargava N., O’Connor A., Gibney E.R., Feeney E.L. (2023). Dairy consumption in adults in China: a systematic review. BMC Nutr..

[19] Tsai C.F., Thomas B., Sudlow C.L. (2013). Epidemiology of stroke and its subtypes in Chinese vs white populations: a systematic review. Neurology.

[20] Chen Z., Chen J., Collins R., Guo Y., Peto R., Wu F. (2011). China Kadoorie Biobank of 0.5 million people: survey methods, baseline characteristics and long-term follow-up. Int. J. Epidemiol..

[21] Du H., Li L., Bennett D., Yang L., Guo Y., Key T.J. (2017). Fresh fruit consumption and all-cause and cause-specific mortality: findings from the China Kadoorie Biobank. Int. J. Epidemiol..

[22] Qin C., Guo Y., Pei P., Du H., Yang L., Chen Y. (2022). The relative validity and reproducibility of food frequency questionnaires in the China Kadoorie Biobank study. Nutrients.

[23] Clarke R., Shipley M, Lewington S., Youngman L., Collins R., Marmot M. (1999). Underestimation of risk associations due to regression dilution in long-term follow-up of prospective studies. Am. J. Epidemiol..

[24] Kakkoura M.G., Du H., Guo Y., Yu C., Yang L., Pei P. (2022). Dairy consumption and risks of total and site-specific cancers in Chinese adults: an 11-year prospective study of 0.5 million people. BMC Med.

[25] Yang G.H., Stroup D.F., Thacker S.B. (1997). National public health surveillance in China: implications for public health in China and the United States, Biomed. Environ. Sci..

[26] Pang Y., Kartsonaki C., Du H., Millwood I.Y., Guo Y., Chen Y. (2019). Physical activity, sedentary leisure time, circulating metabolic markers, and risk of major vascular diseases. Circ. Genom. Precis. Med..

[27] Bragg F., Kartsonaki C., Guo Y., Holmes M., Du H., Yu C. (2021). Circulating metabolites and the development of type 2 diabetes in Chinese adults. Diabetes Care.

[28] Plummer M. (2004). Improved estimates of floating absolute risk. Stat. Med..

[29] Liu Z., Pang S., Li Y., Man Q., Li L., Zhang J. (2016). Consumption status of dairy products in Chinese aged 60 and above in 2010-2012. Wei Sheng Yan Jiu.

[30] Huang F., Wang H., Wang Z., Zhang J., Su C., Du W. (2019). Knowledge, behavior and consumption types of milk and dairy products among the Chinese aged 60 and above in 15 provinces (autonomous regions and municipalities) in 2015. Wei Sheng Yan Jiu.

[31] Wei J., Wang J. (2023). Chinese residents’ knowledge about and behavior towards dairy products: a cross-sectional study. BMC Public Health.

[32] Papier K., Kakkoura M.G., Guo Y., Knuppel A., Pei P., Tong T.Y.N. (2023). Intakes of major food groups in China and UK: results from 100,000 adults in the China Kadoorie biobank and UK biobank. Eur. J. Nutr..

[33] Fuller F., Huang J., Ma H., Rozelle S. (2006). Got milk? The rapid rise of China’s dairy sector and its future prospects. Food Policy.

[34] Huang Y., Wang H., Tian X. (2016). Changing diet quality in China during 2004-2011. Int. J. Environ. Res. Public Health..

[35] Kakkoura M.G., Walters R.G., Clarke R., Chen Z., Du H. (2024). Milk intake, lactase non-persistence and type 2 diabetes risk in Chinese adults. Nat. Metab..

[36] Zong G., Sun Q., Yu D., Zhu J., Sun L., Ye X. (2014). Dairy consumption, type 2 diabetes, and changes in cardiometabolic traits: a prospective cohort study of middle-aged and older Chinese in Beijing and Shanghai. Diabetes Care.

[37] Talaei M., Pan A., Yuan J.M., Koh W.P. (2017). Dairy food intake is inversely associated with risk of hypertension: the Singapore Chinese Health Study. J. Nutr..

[38] Hidayat K., Yu L.-G., Yang J.-R., Zhang X.-Y., Zhou H., Shi Y.-J. (2020). The association between milk consumption and the metabolic syndrome: a cross-sectional study of the residents of Suzhou, China and a meta-analysis. Br. J. Nutr..

[39] Schwingshackl L., Hoffmann G., Schwedhelm C., Kalle-Uhlmann T., Missbach B., Knüppel S. (2016). Consumption of dairy products in relation to changes in anthropometric variables in adult populations: a systematic review and meta-analysis of cohort studies. PLOS ONE.

[40] Li P., Fan C., Lu Y., Qi K. (2016). Effects of calcium supplementation on body weight: a meta-analysis. Am. J. Clin. Nutr..

[41] Auestad N., Layman D.K. (2021). Dairy bioactive proteins and peptides: a narrative review. Nutr. Rev..

[42] McGrane M.M., Essery E., Obbagy J., Lyon J., Macneil P., Spahn J. (2011). Dairy consumption, blood pressure, and risk of hypertension: an evidence-based review of recent literature. Curr. Cardiovasc. Risk Rep..

[43] Vimaleswaran K.S., Zhou A., Cavadino A., Hyppönen E. (2021). Evidence for a causal association between milk intake and cardiometabolic disease outcomes using a two-sample Mendelian Randomization analysis in up to 1,904,220 individuals. Int. J. Obes..

[44] Mendelian Randomization of Dairy Consumption Working Group (2018). Dairy consumption and body mass index among adults: mendelian randomization analysis of 184802 individuals from 25 studies. Clin. Chem..

[45] Ding M., Huang T., Km Bergholdt H., Nordestgaard B.G., Ellervik C., Qi L. (2017). Dairy consumption, systolic blood pressure, and risk of hypertension: Mendelian randomization study. BMJ.

[46] Zhang S., Meng G., Zhang Q., Liu L., Wu H., Gu Y. (2023). Dairy intake and risk of type 2 diabetes: results of a large prospective cohort. Food Funct.

[47] Laouali N., Shah S., MacDonald C.-J., Mahamat-Saleh Y., El Fatouhi D., Mancini F. (2021). BMI in the associations of plant-based diets with type 2 diabetes and hypertension risks in women: the E3N prospective cohort study. J. Nutr..

[48] Papier K., Appleby P.N., Fensom G.K., Knuppel A., Perez-Cornago A., Schmidt J.A. (2019). Vegetarian diets and risk of hospitalisation or death with diabetes in British adults: results from the EPIC-Oxford study, Nutr. Diabetes.

[49] Jensen C.F., Timofeeva M., Berg-Beckhoff G. (2023). Milk consumption and the risk of type 2 diabetes: a systematic review of mendelian randomization studies. Nutr. Metab. Cardiovasc. Dis..

[50] He Y., Wu W., Zheng H.-M., Li P., McDonald D., Sheng H.-F. (2018). Regional variation limits applications of healthy gut microbiome reference ranges and disease models. Nat. Med..

[51] Johansson I., Esberg A., Nilsson L.M., Jansson J.-H., Wennberg P., Winkvist A. (2019). Dairy product intake and cardiometabolic diseases in Northern Sweden: a 33-year prospective cohort study. Nutrients.

[52] Wang X.Y., Liu F.-C., Yang X.-L., Li J.-X., Cao J., Lu X.-F. (2020). Association of cardiovascular diseases with milk intake among general Chinese adults. Chin. Med. J. (Engl)..

[53] Tanno K., Yonekura Y., Okuda N., Kuribayashi T., Yabe E., Tsubota-Utsugi M. (2021). Association between milk intake and incident stroke among Japanese community dwellers: the Iwate-KENCO study. Nutrients.

[54] Tong T.Y.N., Appleby P.N., Bradbury K.E., Perez-Cornago A., Travis R.C., Clarke R. (2019). Risks of ischaemic heart disease and stroke in meat eaters, fish eaters, and vegetarians over 18 years of follow-up: results from the prospective EPIC-Oxford study. BMJ.

[55] Naghshi S., Sadeghi O., Larijani B., Esmaillzadeh A. (2022). High vs. low-fat dairy and milk differently affects the risk of all-cause, CVD, and cancer death: a systematic review and dose-response meta-analysis of prospective cohort studies. Crit. Rev. Food Sci. Nutr..

[56] Wang X.J., Jiang C.Q., Zhang W.S., Zhu F., Jin Y.L., Woo J. (2020). Milk consumption and risk of mortality from all-cause, cardiovascular disease and cancer in older people. Clin. Nutr..

[57] Ronit A., Kirkegaard-Klitbo D.M., Dohlmann T.L., Lundgren J., Sabin C.A., Phillips A.N. (2020). Plasma albumin and incident cardiovascular disease. Arterioscler. Thromb. Vasc. Biol..

[58] Huang T., An Z., Huang Z., Gao W., Hao B., Xu J. (2024). Serum albumin and cardiovascular disease: a Mendelian randomization study. BMC Cardiovasc. Disorders..

[59] Choi J.W., Park J.-S., Lee C.H. (2021). Genetically determined hypoalbuminemia as a risk factor for hypertension: instrumental variable analysis. Sci. Rep..

[60] Dastmalchi L.N., German C.A., Taub P.R. (2023). High density lipoprotein: when to rethink too much of a good thing. Am. J. Prev. Cardiol..

[61] Lawler P.R., Akinkuolie A.O., Chu A.Y., Shah S.H., Kraus W.E., Craig D. (2017). Atherogenic lipoprotein determinants of cardiovascular disease and residual risk among individuals with low low-density lipoprotein cholesterol. J. Am. Heart Assoc..

[62] Dong H., Pi F., Ding Z., Chen W., Pang S., Dong W. (2015). Efficacy of supplementation with B vitamins for stroke prevention: a network meta-analysis of randomized controlled trials. PLOS ONE.

[63] Iso H., Stampfer M.J., Manson J.E., Rexrode K., Hu F., Hennekens C.H. (2001). Prospective study of fat and protein intake and risk of intraparenchymal hemorrhage in women. Circulation.

[64] Luo K., Chen G.-C., Zhang Y., Moon J.-Y., Xing J., Peters B.A. (2024). Variant of the lactase LCT gene explains association between milk intake and incident type 2 diabetes. Nat. Metab..

[65] Miller M., Stone N.J., Ballantyne C., Bittner V., Criqui M.H., Ginsberg H.N. (2011). Triglycerides and cardiovascular disease. Circulation.

