# Supplementary material for: Dairy Consumption and Risk of Cardiometabolic Diseases: A Prospective Cohort Study of the China Kadoorie Biobank
Source: J Nutr. 2026 Jan 31;156(4):101388. doi: 10.1016/j.tjnut.2026.101388 (PMC13084579; doi:10.1016/j.tjnut.2026.101388)

Dairy consumption and risk of cardiometabolic diseases: a prospective cohort study of the China Kadoorie Biobank

Supplementary Material

Tables and Figures

Table of Contents

Members of the China Kadoorie Biobank Collaborative Group 2

eTable 1: Associations of dairy intake (consumers [n=3874] vs non-consumers [n=11,122]) with biochemical markers at baseline survey (2004-2008)........ 3

eTable 2: Adjusted HRs of major cardiometabolic events[a] associated with dairy intake[b]........ 4

eTable 3: Adjusted HRs of major cardiometabolic events associated with dairy intake[a] in sensitivity analyses........ 5

eFigure 1: Proportion of participants and estimated mean dairy intake by sex and survey region among ≥4 days/week dairy consumers at baseline survey (2004-2008). 7

eFigure 2: Proportion of ≥4 days/week dairy consumers by sex, area and age at baseline survey (2004-2008) 8

eFigure 3: Estimated mean dairy intake (g/day) by age in ten regions. 9

eFigure 4: Trend of dairy consumption across the three study surveys........ 10

eFigure 5: Proportion of participants consuming various dairy products at second resurvey (2013-2014). 11

eFigure 6: Adjusted mean values for A) body mass index (BMI), B) random plasma glucose (RPG), C) systolic blood pressure (SBP) and D) diastolic blood pressure (DBP) by the frequency of dairy intake in men (M) and women (F) at baseline survey (2004-2008). 12

eFigure 7: Adjusted mean values for clinical biochemical markers with FDR-corrected P<0.05 by the frequency of dairy intake at baseline survey (2004-2008)........ 13

eFigure 8: Associations of usual dairy intake (g/day) with incidence of A. Diabetes, B. Ischemic heart disease (IHD), C. Acute myocardial infarction (MI), D. Ischemic stroke (IS), E. Intracerebral haemorrhage (ICH) and F. Cardiovascular death, by study area. 14

eFigure 9: Adjusted HRs (95% CIs) for A. ischemic heart disease (IHD), B. Acute myocardial infarction (MI), C. Ischemic stroke (IS), D. Intracerebral haemorrhage (ICH) and E. Cardiovascular death, per 50 g/day of usual dairy intake by region. 15

eFigure 10: Adjusted HRs (95% CIs) for acute MI per 50 g/day of usual dairy intake by baseline characteristics. 16

eFigure 11: Adjusted HRs (95% CIs) for ICH per 50 g/day of usual dairy intake by baseline characteristics........ 17

eFigure 12: Adjusted HRs (95% CIs) for cardiovascular death per 50 g/day of usual dairy intake by baseline characteristics. 18

eFigure 13: Adjusted HRs (95% CIs) for IHD per 50 g/day of usual dairy intake by baseline characteristics. 19

Members of the China Kadoorie Biobank Collaborative Group

International Steering Committee: Junshi Chen, Zhengming Chen (PI), Robert Clarke, Rory Collins, Liming Li (PI), Jun Lv, Richard Peto, Robin Walters.

International Co-ordinating Centre, Oxford: Daniel Avery, Maxim Barnard, Derrick Bennett, Ruth Boxall, Ka Hung Chan, Yiping Chen, Zhengming Chen, Charlotte Clarke, Johnathan Clarke; Robert Clarke, Huaidong Du, Ahmed Edris Mohamed, Hannah Fry, Simon Gilbert, Prapthi Harish, Pek Kei Im, Andri Iona, Maria Kakkoura, Christiana Kartsonaki, Kshitij Kolhe, Hubert Lam, Kuang Lin, James Liu, Mohsen Mazidi, Iona Millwood, Sam Morris, Qunhua Nie, Alfred Pozarickij, Maryam Rahmati, Paul Ryder, Dan Schmidt, Becky Stevens, Iain Turnbull, Robin Walters, Baihan Wang, Lin Wang, Neil Wright, Ling Yang, Xiaoming Yang, Pang Yao.

National Co-ordinating Centre, Beijing: Jun Lv, Canqing Yu, Dianjianyi Sun, Yuanjie Pang, Can Hou, Qingmei Xia, Chao Liu, Pei Pei, Lang Pan, Xiao Han, Honglu Bian, Xinxin Chen.

10 Regional Co-ordinating Centres:

Qingdao CDC: Zengchang Pang, Ruqin Gao, Shanpeng Li, Haiping Duan, Shaojie Wang, Yongmei Liu, Ranran Du, Liang Cheng, Xiaocao Tian, Hua Zhang. **Licang CDC:** Dan Hu, Xiaoyan Zheng, Yujie Wang. **Heilongjiang Provincial CDC:** Wei Sun, Shichun Yan, Xiaoming Cui. **Nangang CDC:** Chi Wang, Zhenyuan Wu, Lishun Zhai, Zhaoxi Pang, Shiwen Dong. **Hainan Provincial CDC:** Huiming Luo, Jinyan Chen, Bin He, Dingwei Sun, Xingren Wang, Tingting Ou. **Meilan CDC:** Xiangyang Zheng, Dewei Zheng, Shuai Yang, Yilei Li, Lihui Li, Xingjiao Chen. **Jiangsu Provincial CDC:** Jinyi Zhou, Ran Tao, Jian Su, Xikang Fan, Zongming Cheng, Yuxiao Huang. **Suzhou CDC:** Yan Lu, Yujie Hua, Li Xing, Shuxian Wang, Jianrong Jin, Juping Ma, Jinchao Liu, Kaifei Zhu, Hongfu Ren, Xingfeng Shen. **Guangxi Provincial CDC:** Ge Zhong, Wei Mao, Zhenzhen Lu, Ling He. **Liuzhou CDC:** Lifang Zhou, Changping Xie, Jian Lan, Tingping Zhu, Jinxue Tan, Liuping Wei, Liyuan Zhou, Sisi Wang. **Sichuan Provincial CDC:** Xianping Wu, Ningmei Zhang, Xiaofang Chen, Xiaoyu Chang, Zhuo Wang, Yujin He. **Pengzhou CDC:** Mingqiang Yuan, Xia Wu, Xiaofang Chen, Zhaodong Wang, Qiang Sun, Yang Lin. **Gansu Provincial CDC:** Faqing Chen, Xiaolan Ren, Lijun Chang, Feiming Zhong. **Maiji CDC:** Jianjun Feng, Weijie Hu, Xiaofang Zhang, Yalin Chen, Fei Wang, Jun Wang. **Henan Provincial CDC:** Linqi Diao, Wanshen Guo, Zhiwei Han, Dongyang Zhao, Dengjun Zhu, Kai Kang, Shixian Feng, Huizi Tian, Yali Yan, Bing Han, Li Gao, Shaofang Li, Huafei Feng, Wei Tang. **Huixian CDC:** Xiaolin Li, Huarong Sun, Xiaocong Zhao, Ying Li, Chen Hu, Pan He, Xukui Zhang, Yuanyuan Jin, Hesheng Zhang. **Zhejiang Provincial CDC:** Min Yu, Ruying Hu, Hao Wang, Weiwei Gong, Jieming Zhong, Meng Wang, Chunxiao Xu, Keqing Gong. **Tongxiang CDC**: Hao Xu, Yuan Cao, Kaixu Xie, Lingli Chen, Xiaomei Tu,Chen Chen. **Hunan Provincial CDC:** Xiaojun Li, Li Yin, Huilin Liu, Yuan Liu, Yi Liu, Lei Yin, Xian Xie, Jing Wang. **Liuyang CDC:** Bo Xiao, Pingsheng Lou, Yuan Peng, Libo Zhang, Chan Qu, Qili Jiang, Yanling Chen, Yan Zhao.

eTable 1: Associations of dairy intake (consumers [n=3874] vs non-consumers [n=11,122]) with biochemical markers at baseline survey (2004-2008)

Biochemical markers	Unit	Coefficient	Standard error	FDR-adjusted *P* value
**Uric acid**	umoll/L	0.06	0.02	0.00
**Total triglycerides**	mmoll/L	0.07	0.02	0.00
**Albumin**	g/L	0.06	0.02	0.02
**Apolipoprotein A1**	mg/dL	0.06	0.02	0.02
**Gamma glutamyl transferase**	u/L	-0.03	0.01	0.06
**25-hydroxyvitamin D**	ng/ml	-0.06	0.03	0.06
**HDL cholesterol**	mmoll/L	0.04	0.02	0.06
**Fibrinogen**	g/L	0.05	0.03	0.14
**Total cholesterol**	mmoll/L	0.03	0.02	0.17
**High sensitivity CRP**	mg/L	-0.03	0.02	0.35
**Non-HDL cholesterol**	mmoll/L	0.02	0.02	0.44
**Alanine aminotranferase**	u/L	0.02	0.02	0.44
**Lipoprotein (a)**	nmol/L	-0.02	0.02	0.46
**Aspartate aminotransferase**	u/L	0.02	0.02	0.58
**Creatinine**	umoll/L	-0.01	0.01	0.73
**Non-HDL/Total cholesterol ratio**	-	-0.01	0.02	0.73
**Apolipoprotein B**	mg/dL	0.01	0.02	0.73
**Cystatin C**	mg/L	0.01	0.02	0.76
**LDL cholesterol**	mmoll/L	0.00	0.02	0.89

Values of circulating biochemical markers were standardized to have a standard deviation of 1 and analysis was adjusted for age, age2, sex, region, fasting time, education, income, smoking, alcohol intake, total physical activity, family history of cardiovascular disease (CVD), consumption of fresh fruit, red meat, poultry, fish and eggs and body mass index (BMI). Inverse probability of sampling weighting was used to account for the ascertainment status of the participants. *P* values were corrected using the Benjamini-Hochberg false discovery rate (FDR) method at 0.05. Participants with prevalent CVD, diabetes or cancer were excluded from the analysis.

CRP: C-reactive protein; HDL: high density lipoprotein; LDL: low density lipoprotein.

eTable 2: Adjusted HRs of major cardiometabolic events[a] associated with dairy intake[b]

Cardiometabolic disease type	Dairy products intake				*P* trend	HR (95% CI) per 50 g/day of usual dairy intake
	Never/rarely	Monthly	1-3 days/week	≥4 days/week		
**Diabetes**						
No. of events	12,855	1744	1347	1718		
Main model[c]	1.00 (0.97-1.03)	1.04 (0.99-1.09)	1.04 (0.98-1.10)	1.00 (0.95-1.06)	0.50	1.01 (0.98-1.04)
Main model + SBP[d]	1.00 (0.97-1.03)	1.05 (1.00-1.10)	1.05 (1.00-1.11)	1.02 (0.97-1.08)	0.16	1.02 (0.99-1.05)
**Heart disease**						
**IHD**						
No. of events	20,781	4031	3485	5649		
Main model[c]	1.00 (0.98-1.02)	1.05 (1.02-1.09)	1.09 (1.05-1.13)	1.11 (1.08-1.15)	<0.0001	1.06 (1.04-1.08)
Main model + SBP[d]	1.00 (0.98-1.02)	1.06 (1.03-1.09)	1.10 (1.06-1.14)	1.13 (1.10-1.16)	<0.0001	1.07 (1.05-1.09)
**Acute MI**						
No. of events	2699	472	271	446		
Main model[c]	1.00 (0.95-1.05)	1.03 (0.94-1.13)	0.92 (0.81-1.04)	0.88 (0.80-0.98)	0.04	0.93 (0.88-0.99)
Main model + SBP[d]	1.00 (0.95-1.05)	1.04 (0.95-1.14)	0.93 (0.82-1.05)	0.90 (0.81-1.00)	0.08	0.94 (0.88-1.00)
**Stroke**						
**IS**						
No. of events	22,332	4208	2883	4247		
Main model[c]	1.00 (0.98-1.02)	1.05 (1.01-1.08)	1.00 (0.96-1.04)	0.96 (0.93-0.99)	0.13	0.98 (0.96-1.00)
Main model + SBP[d]	1.00 (0.98-1.02)	1.06 (1.03-1.10)	1.03 (0.99-1.06)	1.00 (0.96-1.03)	0.61	1.00 (0.98-1.02)
**ICH**						
No. of events	5693	724	357	417		
Main model[c]	1.00 (0.96-1.04)	0.92 (0.86-0.99)	0.86 (0.77-0.95)	0.69 (0.62-0.76)	<0.0001	0.83 (0.79-0.88)
Main model +SBP[d]	1.00 (0.96-1.04)	0.96 (0.90-1.03)	0.92 (0.83-1.03)	0.77 (0.69-0.85)	<0.0001	0.88 (0.84-0.93)
**Cardiovascular death**						
No. of events	10,030	1465	693	1026		
Main model[c]	1.00 (0.97-1.03)	0.99 (0.94-1.04)	0.89 (0.82-0.96)	0.82 (0.77-0.87)	<0.0001	0.90 (0.87-0.94)
Main model +SBP[d]	1.00 (0.97-1.03)	1.02 (0.97-1.08)	0.94 (0.87-1.01)	0.88 (0.83-0.94)	0.002	0.94 (0.91-0.98)

[a]Events of ischemic heart disease (IHD), acute myocardial infarction (MI), intracerebral haemorrhage (ICH) and ischemic stroke (IS) were censored for each other.
[b]Analysis was performed among 461,047 participants with no prior self-reported history of cardiovascular disease (CVD), diabetes or cancer at baseline.
[c]Main model: Analysis was stratified by age-at-risk (continuous variable), sex (dichotomous variable) and individual regions (ten regions) and were adjusted for baseline age (continuous), education (four categories), income (four categories), smoking (four categories), alcohol consumption (four categories), total physical activity (continuous variable), family history of CVD (dichotomous variable), consumption of fresh fruit (five categories), red meat (four categories), poultry (three categories), fish (four categories) and eggs (four categories) and body mass index (BMI) (continuous variable).
[d]Main model + SBP: as for main model, additionally adjusted for systolic blood pressure (SBP) (continuous variable).
CI: confidence interval; HR: hazard ratio.

eTable 3: Adjusted HRs of major cardiometabolic events associated with dairy intake[a] in sensitivity analyses

Cardiometabolic disease type	No. of events	HR (95% CI) per 50 g/day of usual dairy intake
**Main model[b]**		
Diabetes	17664	1.01 (0.98-1.04)
IHD	33946	1.06 (1.04-1.08)
Acute MI	3888	0.93 (0.88-0.99)
IS	33670	0.98 (0.96-1.00)
ICH	7191	0.83 (0.79-0.88)
Cardiovascular death	13214	0.90 (0.87-0.94)
**Additional adjustment for rice intake**		
Diabetes	17664	1.01 (0.98-1.04)
IHD	33946	1.06 (1.04-1.08)
Acute MI	3888	0.93 (0.87-0.99)
IS	33670	0.98 (0.96-1.00)
ICH	7191	0.83 (0.79-0.88)
Cardiovascular death	13214	0.90 (0.87-0.94)
**Additional adjustment for wheat intake**		
Diabetes	17664	1.01 (0.98-1.04)
IHD	33946	1.06 (1.04-1.08)
Acute MI	3888	0.93 (0.87-0.99)
IS	33670	0.98 (0.96-1.00)
ICH	7191	0.83 (0.79-0.88)
Cardiovascular death	13214	0.90 (0.87-0.94)
**Additional adjustment for coarse grain foods intake**		
Diabetes	17664	1.01 (0.98-1.04)
IHD	33946	1.06 (1.04-1.08)
Acute MI	3888	0.93 (0.87-0.99)
IS	33670	0.98 (0.96-1.00)
ICH	7191	0.83 (0.79-0.88)
Cardiovascular death	13214	0.90 (0.87-0.94)
**Additional adjustment for fresh vegetables intake**		
Diabetes	17664	1.01 (0.98-1.04)
IHD	33946	1.06 (1.04-1.08)
Acute MI	3888	0.93 (0.88-0.99)
IS	33670	0.98 (0.96-1.00)
ICH	7191	0.83 (0.79-0.88)
Cardiovascular death	13214	0.90 (0.87-0.94)
**Additional adjustment for preserved vegetables intake**		
Diabetes	17664	1.01 (0.98-1.04)
IHD	33946	1.06 (1.04-1.08)
Acute MI	3888	0.93 (0.88-0.99)
IS	33670	0.98 (0.96-1.00)
ICH	7191	0.83 (0.79-0.88)
Cardiovascular death	13214	0.90 (0.87-0.94)
**Additional adjustment for soy intake**		
Diabetes	17664	1.01 (0.98-1.04)
IHD	33946	1.06 (1.04-1.08)
Acute MI	3888	0.93 (0.88-0.99)
IS	33670	0.98 (0.96-1.00)
ICH	7191	0.83 (0.78-0.87)
Cardiovascular death	13214	0.90 (0.87-0.93)
**Additional adjustment for standing height**		
Diabetes	17664	1.01 (0.98-1.04)
IHD	33946	1.06 (1.04-1.08)
Acute MI	3888	0.93 (0.88-0.99)
IS	33670	0.98 (0.96-1.00)
ICH	7191	0.84 (0.79-0.89)
Cardiovascular death	13214	0.91 (0.88-0.94)
**Additional adjustment for WC**		
Diabetes	17664	1.01 (0.98-1.04)
IHD	33946	1.06 (1.04-1.08)

Acute MI	3888	0.93 (0.88-0.99)
IS	33670	0.98 (0.96-1.00)
ICH	7191	0.83 (0.79-0.88)
Cardiovascular death	13214	0.90 (0.87-0.94)
**Additional adjustment for RPG[c]**		
Diabetes	17118	1.01 (0.98-1.04)
IHD	33558	1.06 (1.04-1.08)
Acute MI	3835	0.93 (0.88-0.99)
IS	33320	0.98 (0.96-1.00)
ICH	7033	0.83 (0.79-0.88)
Cardiovascular death	12982	0.90 (0.87-0.94)
**Exclusion of the first 2 years of follow-up**		
Diabetes	16693	1.00 (0.97-1.03)
IHD	30463	1.05 (1.03-1.07)
Acute MI	3417	0.91 (0.86-0.98)
IS	31083	0.97 (0.95-0.99)
ICH	6199	0.83 (0.78-0.88)
Cardiovascular death	12001	0.89 (0.86-0.93)

[a]Analysis was performed among 461,046 participants with no prior self-reported history of cardiovascular disease (CVD), diabetes or cancer at baseline and events of ischemic heart disease (IHD), acute myocardial infarction (MI), intracerebral haemorrhage (ICH) and ischemic stroke (IS) were censored for each other.

[b]Analysis was stratified by age-at-risk (continuous variable), sex (dichotomous variable) and individual regions (ten regions) and were adjusted for baseline age (continuous), education (four categories), income (four categories), smoking (four categories), alcohol consumption (four categories), total physical activity (continuous variable), family history of CVD or diabetes (dichotomous variable), consumption of fresh fruit (five categories), red meat (four categories), poultry (three categories), fish (four categories) and eggs (four categories) and body mass index (BMI) (continuous).

[c]Values for random plasma glucose (RPG) were missing for 7961 participants.

HR: hazard ratio; WC: waist circumference.

eFigure 1: Proportion of participants and estimated mean dairy intake by sex and survey region among ≥4 days/week dairy consumers at baseline survey (2004-2008). Values were adjusted for age at baseline (eight categories). Bars with light grey indicate percentage of regular consumers in urban areas, while white bars indicate regular consumers in rural areas (y-axis on the left). The point graph indicates the estimated mean dairy intake (g/day) (y-axis on the right). Mean usual dairy intake (g/day) was estimated using the daily amount collected at second resurvey and taking into account the consumption frequency change from baseline to second resurvey. Analysis was performed among 49,246 participants with regular dairy consumption (≥4 days/week) with no prior self-reported history of cardiovascular disease (CVD), diabetes or cancer at baseline.

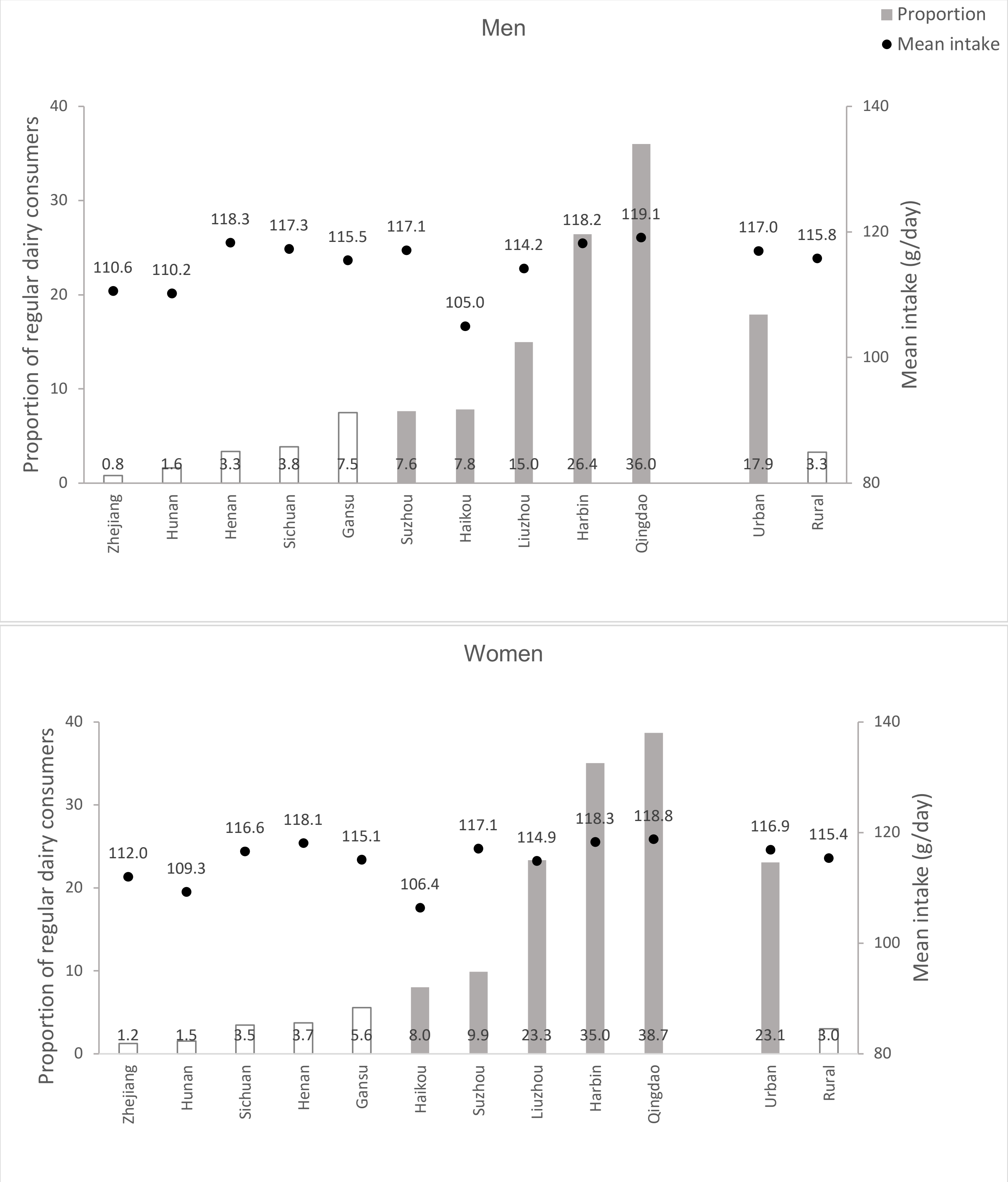

eFigure 2: Proportion of ≥4 days/week dairy consumers by sex, area and age at baseline survey (2004-2008). Analysis was performed among 461,046 participants with no prior self-reported history of cardiovascular disease (CVD), diabetes or cancer at baseline.

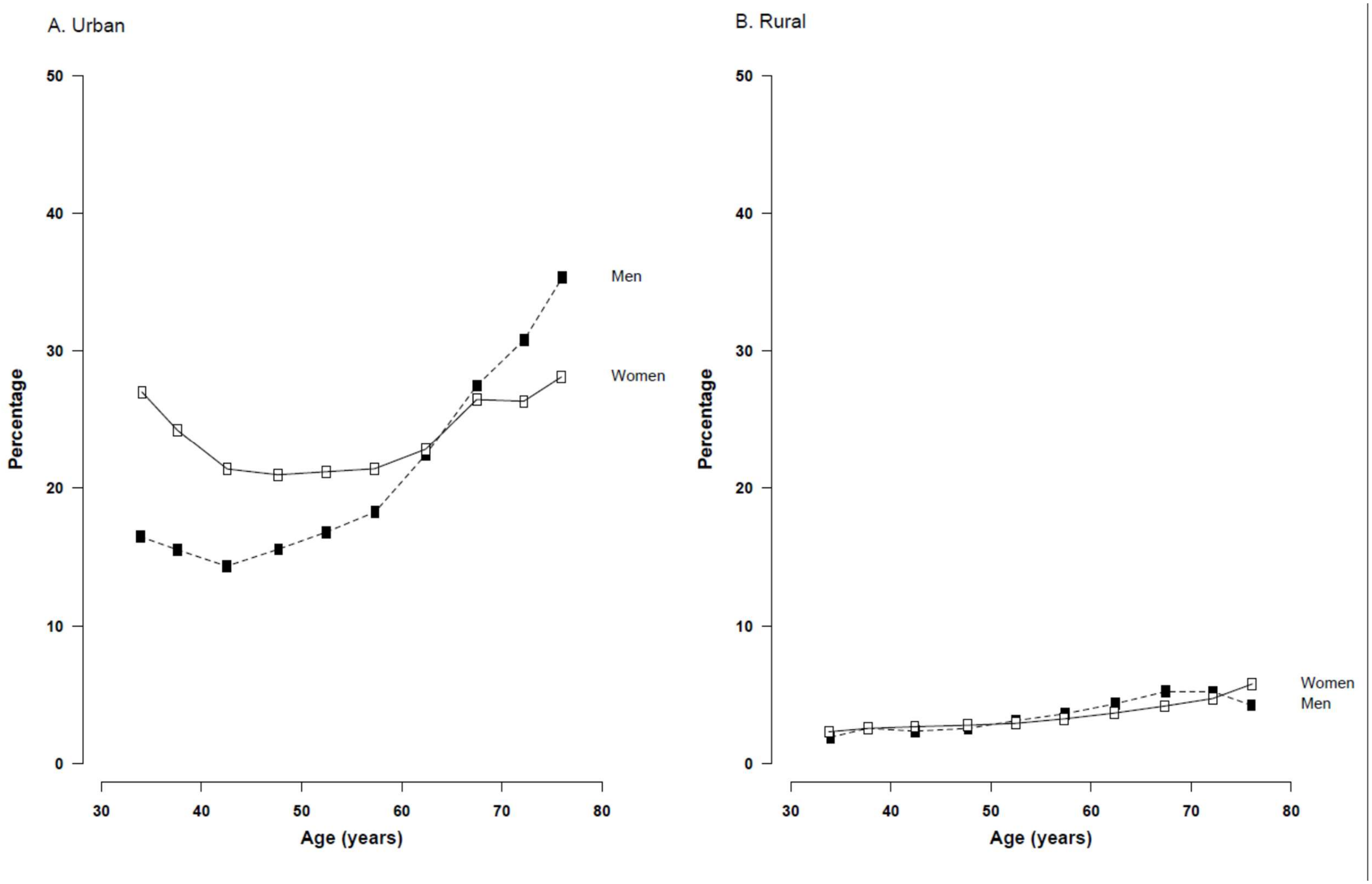

eFigure 3: Estimated mean dairy intake (g/day) by age in ten regions. Mean usual dairy intake (g/day) was estimated using the daily amount collected at second resurvey and taking into account the consumption frequency change from baseline to second resurvey. Analysis was performed among 461,046 participants with no prior self-reported history of cardiovascular disease (CVD), diabetes or cancer at baseline. F: women; M: men.

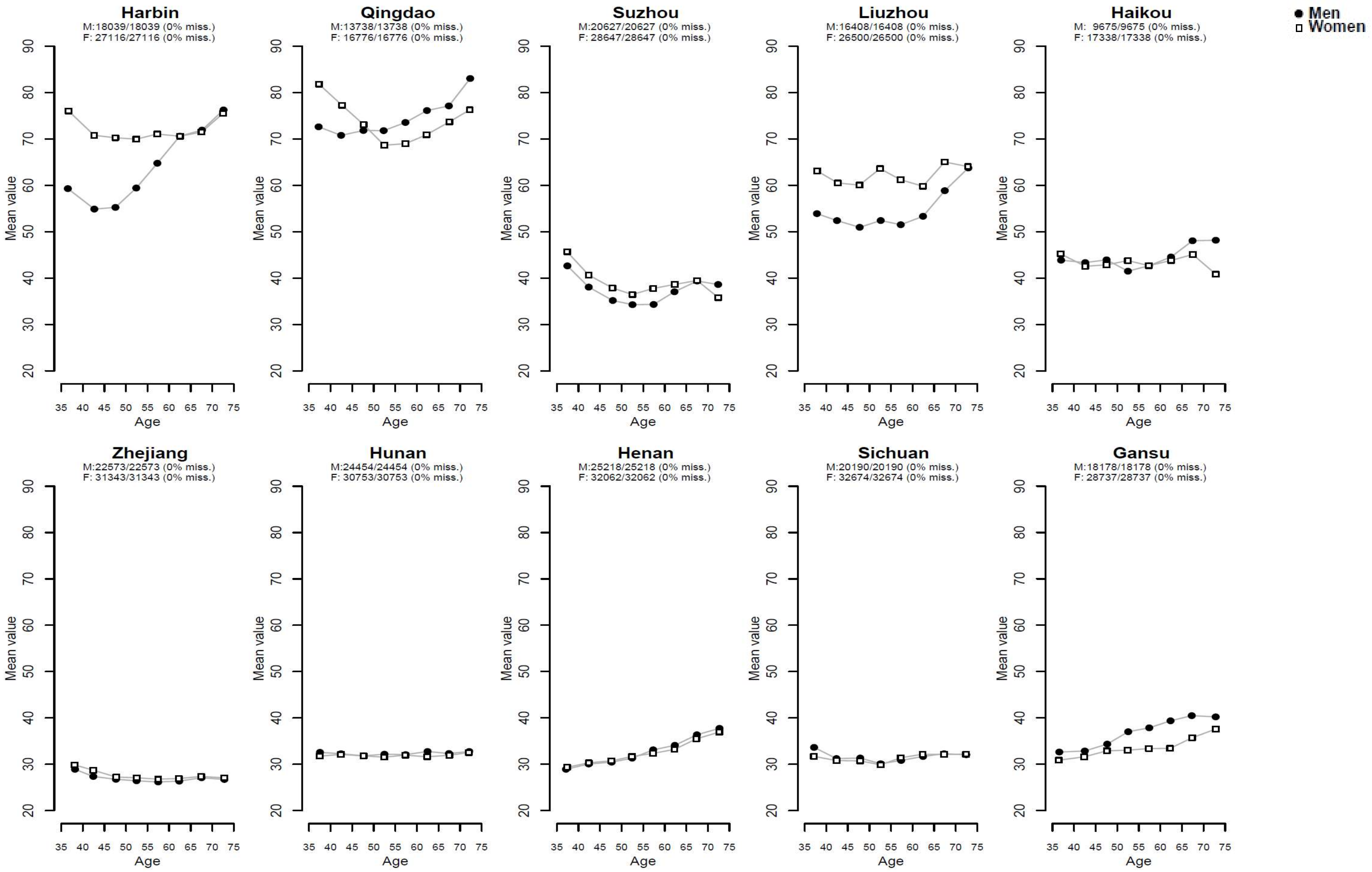

eFigure 4: Trend of dairy consumption across the three study surveys. Bars indicate percentage of participants consuming dairy products at different survey with no prior self-reported history of CVD, diabetes or cancer at baseline. Different grey shades in each bar represent different frequency of dairy consumption.

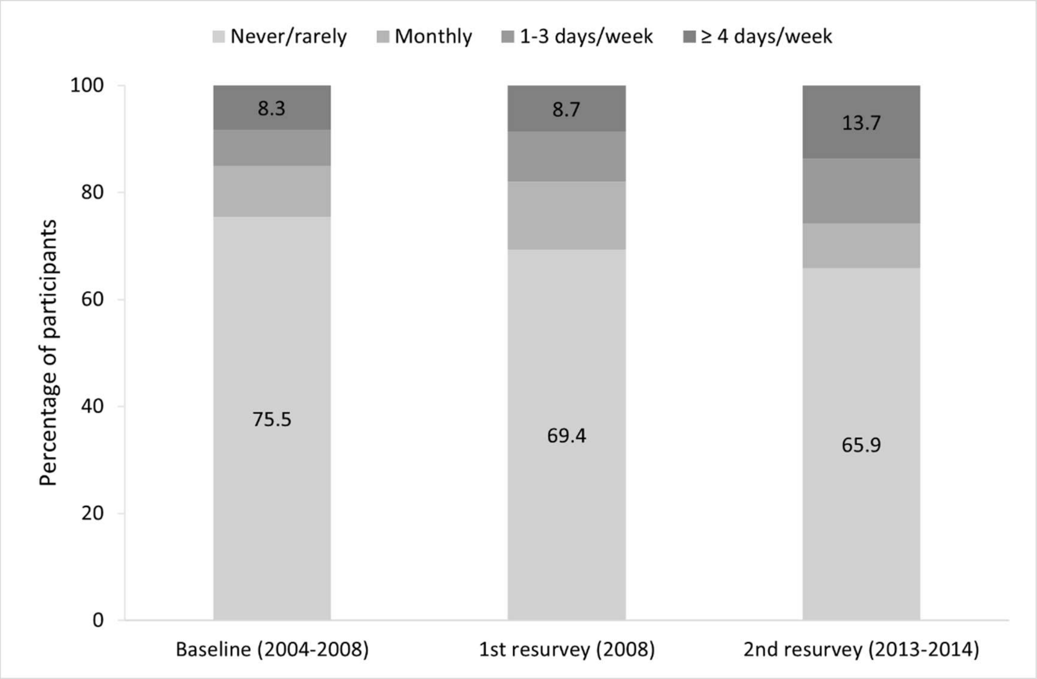

eFigure 5: Proportion of participants consuming various dairy products at second resurvey (2013-2014). The pie chart indicates percentage of participants consuming dairy products (consumption ranging from daily-monthly) among 7071 dairy consumers (consumers for more than one dairy product) without cardiovascular disease (CVD), diabetes, or cancer at either baseline or second resurvey.

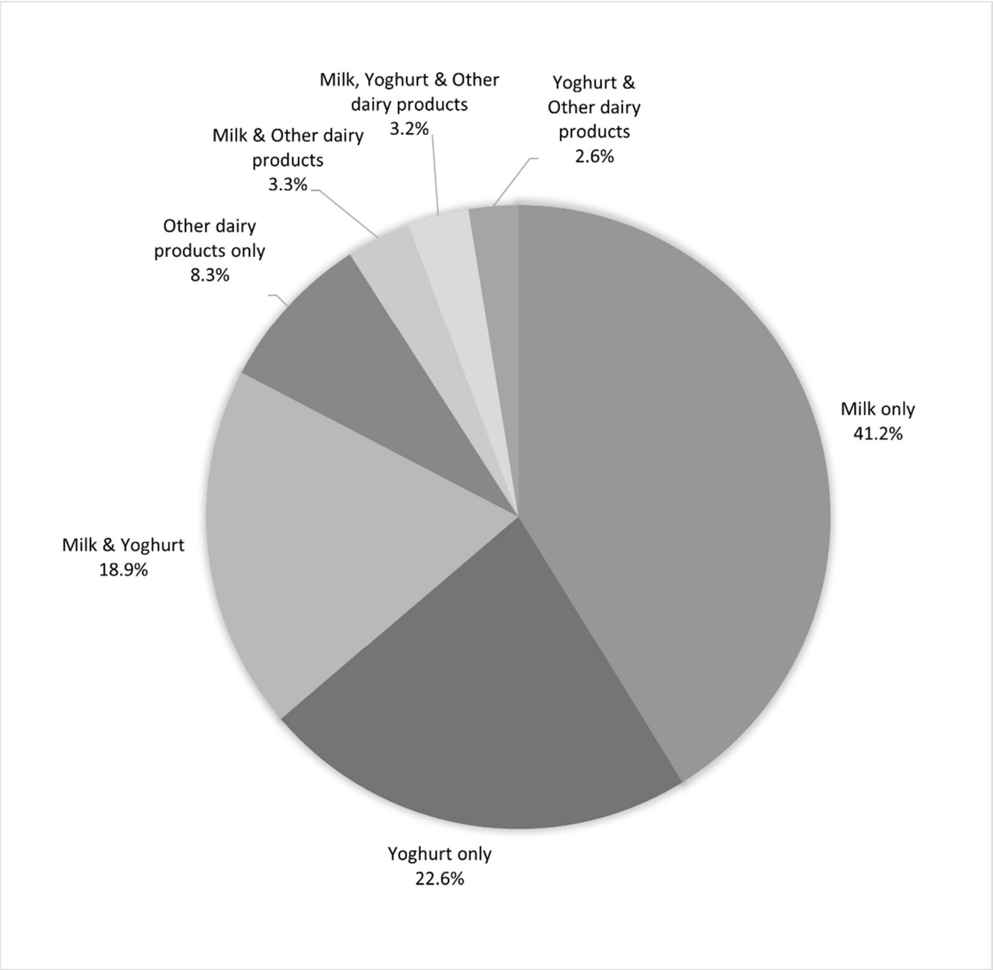

eFigure 6: Adjusted mean values for A) body mass index (BMI), B) random plasma glucose (RPG), C) systolic blood pressure (SBP) and D) diastolic blood pressure (DBP) by the frequency of dairy intake in men (M) and women (F) at baseline survey (2004-2008). Mean values for BMI were adjusted for age, region, education, income, smoking, alcohol intake, total physical activity, family history of cardiovascular disease (CVD) and consumption of fresh fruit, red meat, poultry, fish and eggs. Mean values for RPG, SBP and DBP were additionally adjusted for BMI and waist circumference. Participants with prevalent CVD, diabetes or cancer were excluded from the analysis. Values for RPG were missing for 7961 participants.

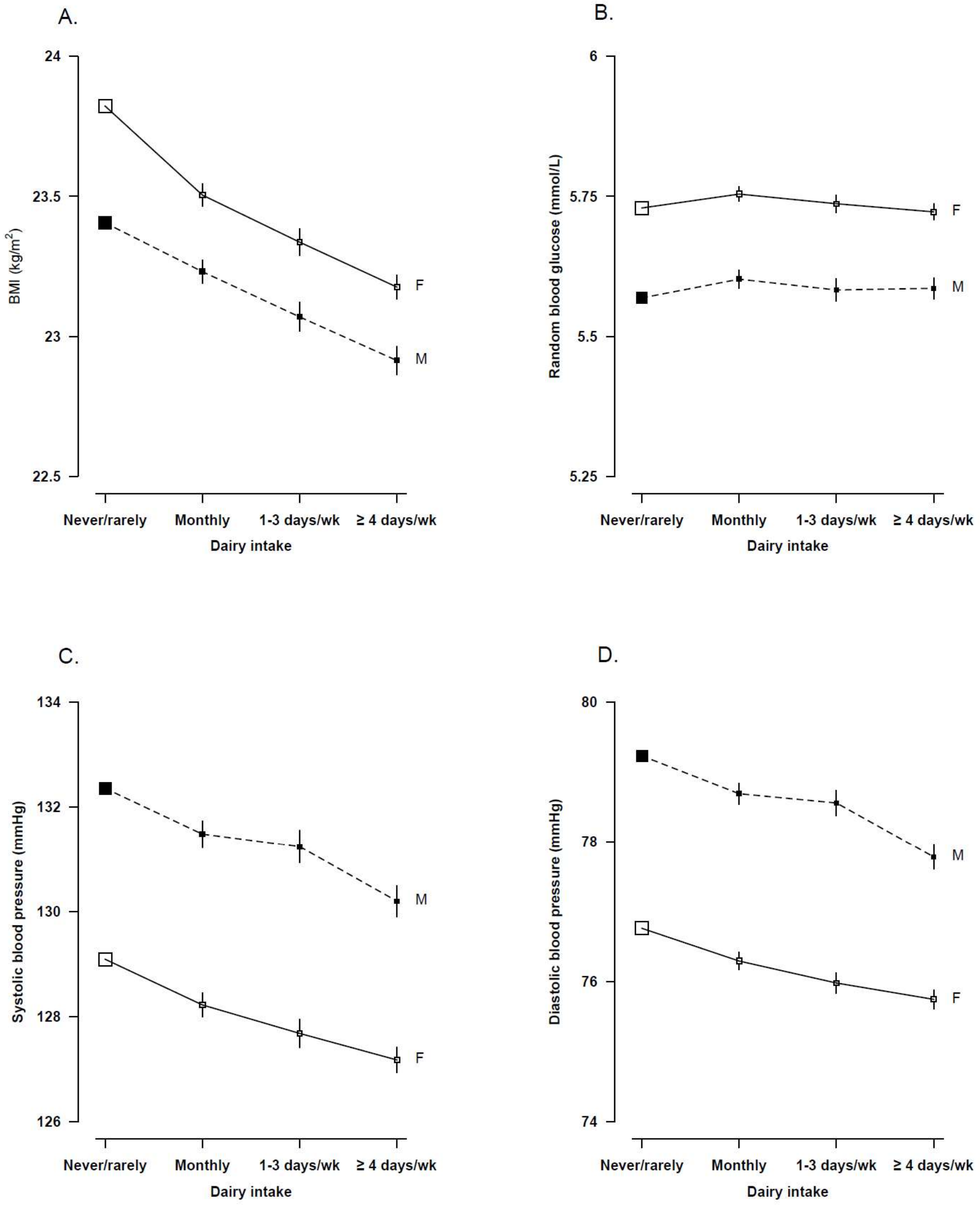

eFigure 7: Adjusted mean values for clinical biochemical markers with FDR-corrected P<0.05 by the frequency of dairy intake at baseline survey (2004-2008). A) total glycerides (n=14,924), B) apolipoprotein A1 (n=14,924), C) albumin (n=14,020), and D) uric acid (n=13,862). Values of circulating biochemical markers were standardized to have a standard deviation of 1 and mean values were adjusted for age, age^2, sex, region, fasting time, education, income, smoking, alcohol intake, total physical activity, family history of cardiovascular disease (CVD), consumption of fresh fruit, red meat, poultry, fish and eggs and body mass index (BMI). Inverse probability of sampling weighting was used to account for the ascertainment status of the participants. Participants with prevalent CVD, diabetes or cancer were excluded from the analysis.

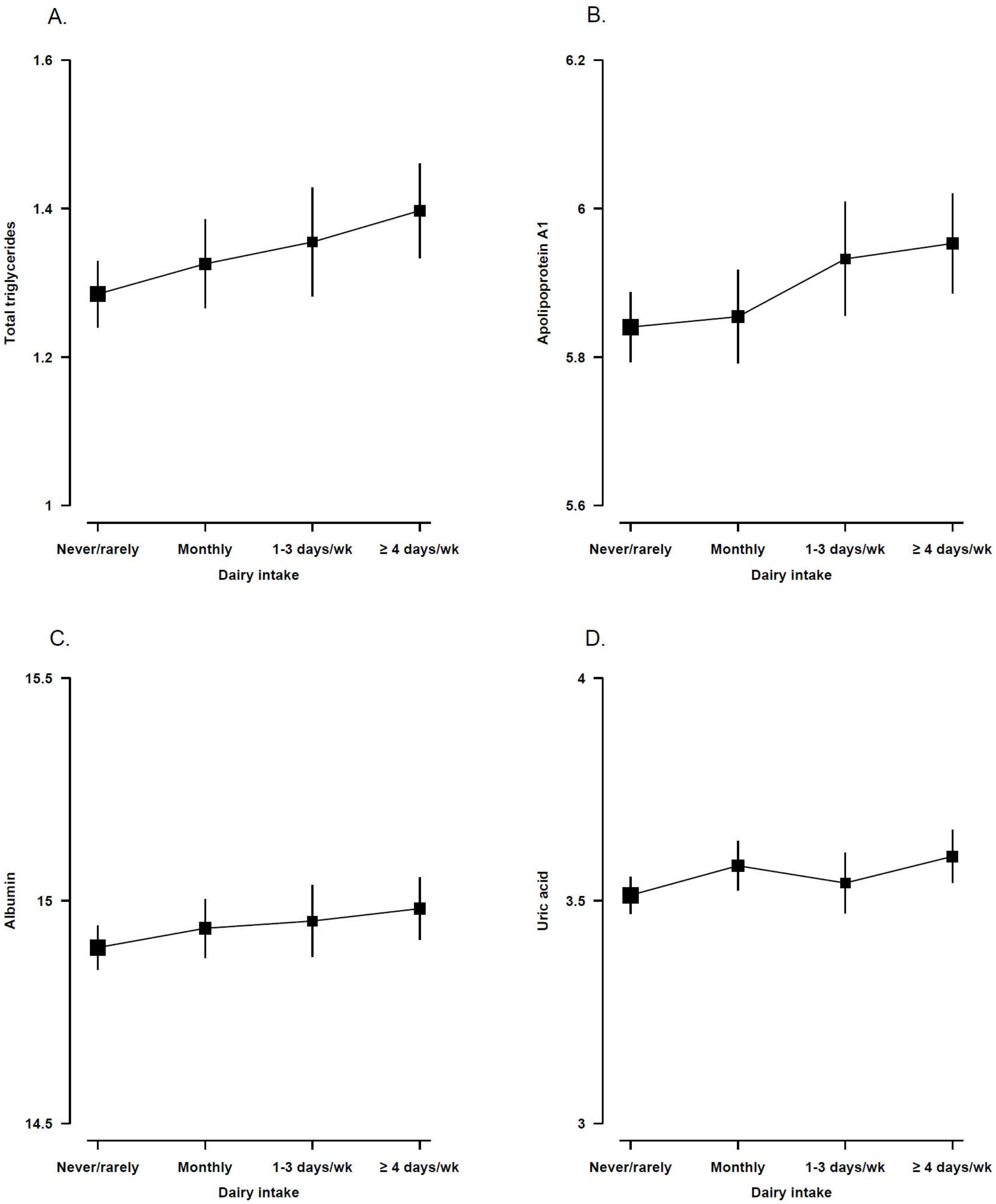

eFigure 8: Associations of usual dairy intake (g/day) with incidence of A. Diabetes, B. Ischemic heart disease (IHD), C. Acute myocardial infarction (MI), D. Ischemic stroke (IS), E. Intracerebral haemorrhage (ICH) and F. Cardiovascular death, by study area. Events of IHD, acute MI, IS and ICH were censored for each other. Analysis was stratified by age-at-risk, sex and region and were adjusted for baseline age (continuous), education (four categories), income (four categories), smoking (four categories), alcohol consumption (four categories), total physical activity (continuous variables), family history of cardiovascular disease (CVD) or diabetes (dichotomous), consumption of fresh fruit (five categories), red meat (four categories), poultry (three categories), fish (four categories) and eggs (four categories) and body mass index (BMI) (continuous). The y axis was plotted on a $\log_e$ scale with the lowest intake group as a reference category. The squares represent hazard ratios (HRs) with the size being inversely proportional to the variance of the $\log_e$ of HR and the vertical lines represent 95% confidence intervals (CIs). The numbers next to the squares are point estimates for HRs. Solid squares represent rural areas and open squares represent urban areas.

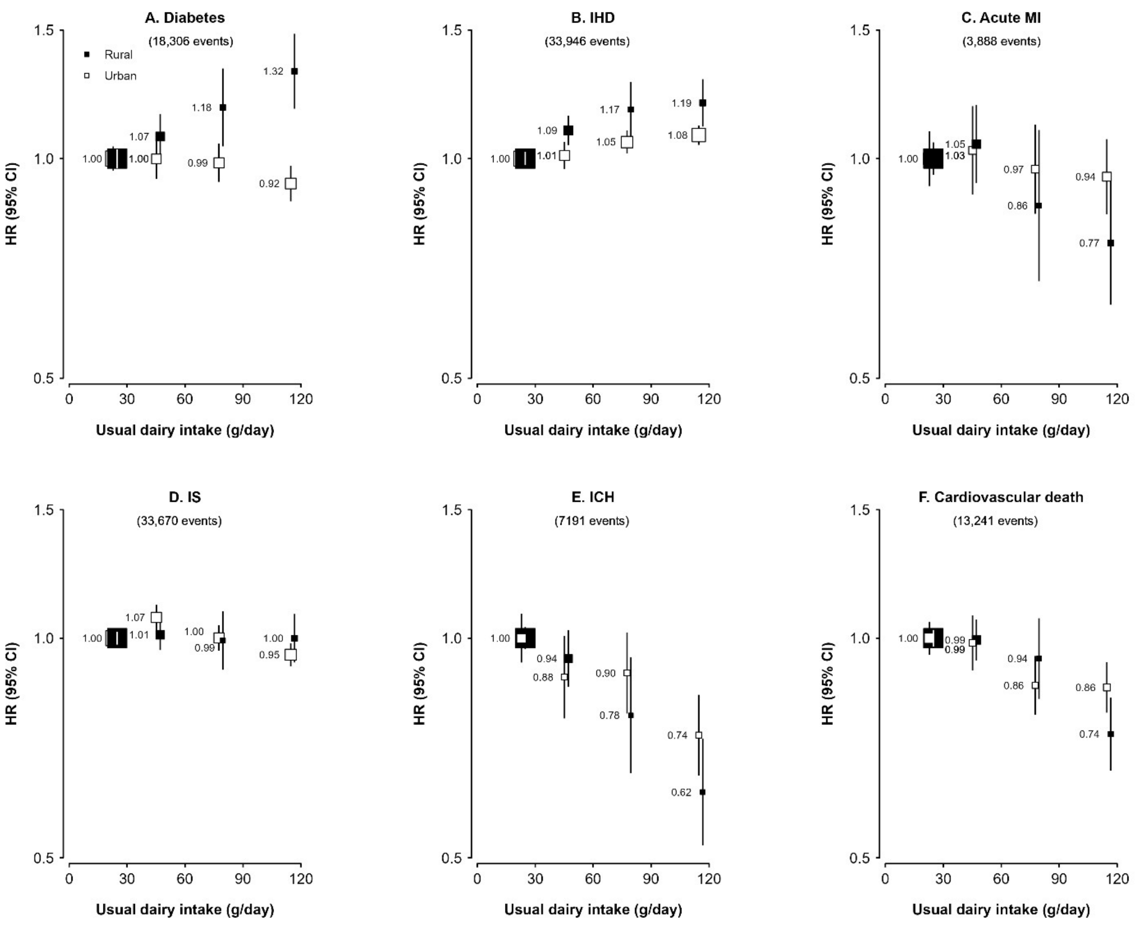

eFigure 9: Adjusted HRs (95% CIs) for A. ischemic heart disease (IHD), B. Acute myocardial infarction (MI), C. Ischemic stroke (IS), D. Intracerebral haemorrhage (ICH) and E. Cardiovascular death, per 50 g/day of usual dairy intake by region. Events of IHD, acute MI, IS and ICH were censored for each other. Analysis was stratified by age-at-risk (continuous variable), sex (dichotomous variable) and region (ten regions) and were adjusted for baseline age (continuous), education (four categories), income (four categories), smoking (four categories), alcohol consumption (four categories), total physical activity (continuous variable), family history of cardiovascular disease (CVD) (dichotomous variable), consumption of fresh fruit (five categories), red meat (four categories), poultry (three categories), fish (four categories) and eggs (four categories) and body mass index (BMI) (continuous). Overall hazard ratio (HR) per 50 g/day usual dairy intake after correcting for regression dilution bias. Black squares represent HRs (size is inversely proportional to the variance of the $\log_e$ of HR); horizontal lines represent 95% confidence intervals (CIs); white diamonds represent overall HRs (95% CIs); 'No of events' refers to the number of incident CVD events or deaths in each group; the subscript numbers in the chi-square values represent the degrees of freedom. Het: heterogeneity.

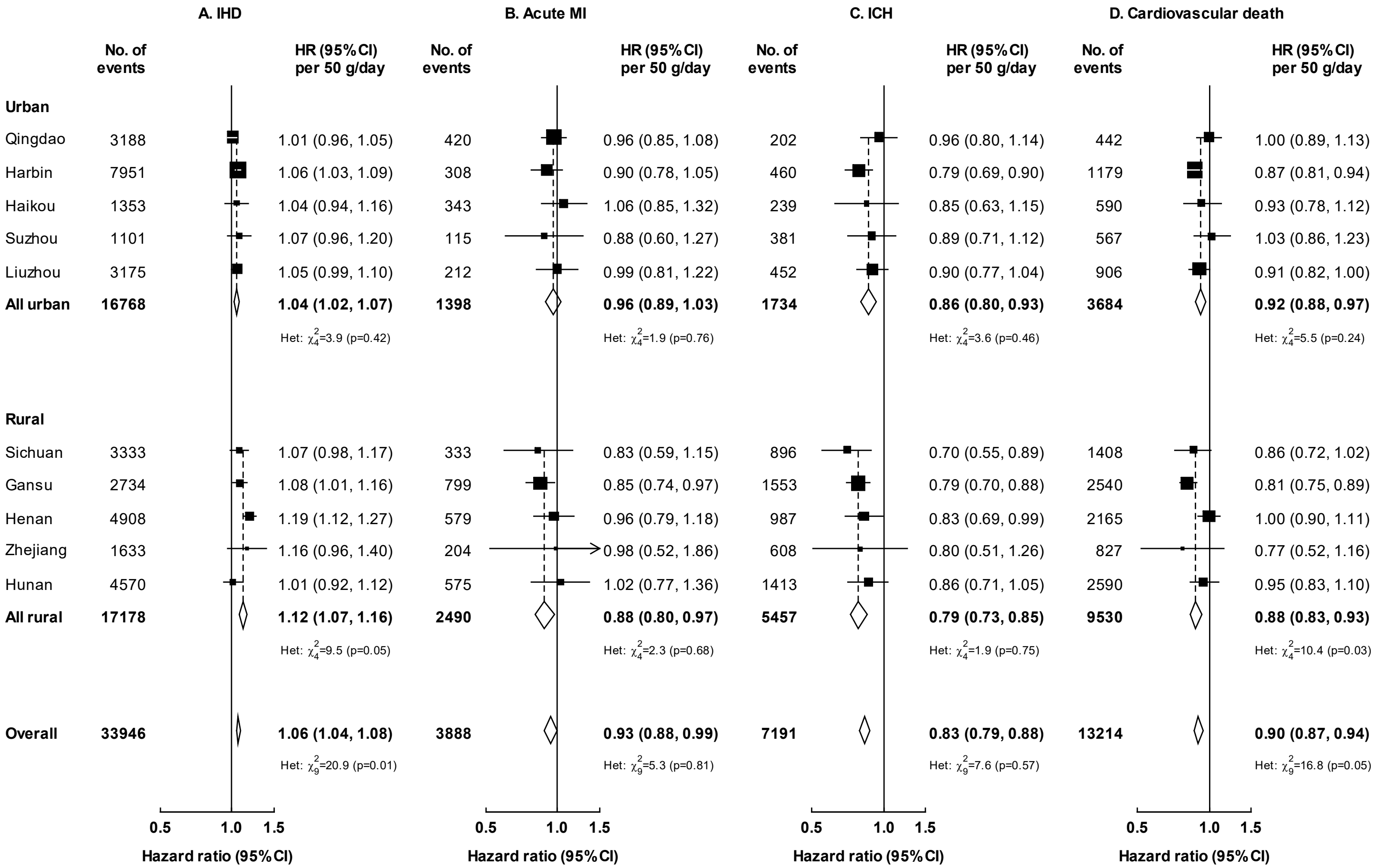

eFigure 10: Adjusted HRs (95% CIs) for acute MI per 50 g/day of usual dairy intake by baseline characteristics. Events of ischemic heart disease (IHD), acute myocardial infarction (MI), intracerebral haemorrhage (ICH) and ischemic stroke (IS) were censored for each other. Analysis was stratified by age-at-risk (continuous variable), sex (dichotomous variable) and region (ten regions) and were adjusted for baseline age (continuous), education (four categories), income (four categories), smoking (four categories), alcohol consumption (four categories), total physical activity (continuous variable), family history of cardiovascular disease (CVD) (dichotomous variable), consumption of fresh fruit (five categories), red meat (four categories), poultry (three categories), fish (four categories) and eggs (four categories) and body mass index (BMI) (continuous). [a]Sex-specific physical activity assessed as metabolic equivalent of task (MET) (h/day). [b]Overall hazard ratio (HR) per 50 g/day usual dairy intake after correcting for regression dilution bias. [c]Overall HR per 50 g/day baseline dairy intake before correcting for regression dilution bias. Black squares, HRs (size is inversely proportional to the variance of the $\log_e$ of HR); horizontal lines represent 95% confidence intervals (CIs); white diamonds, overall HRs (95% CIs); 'No of events' refers to the number of incident CVD events or deaths in each group; the subscript numbers in the chi-square values represent the degrees of freedom.

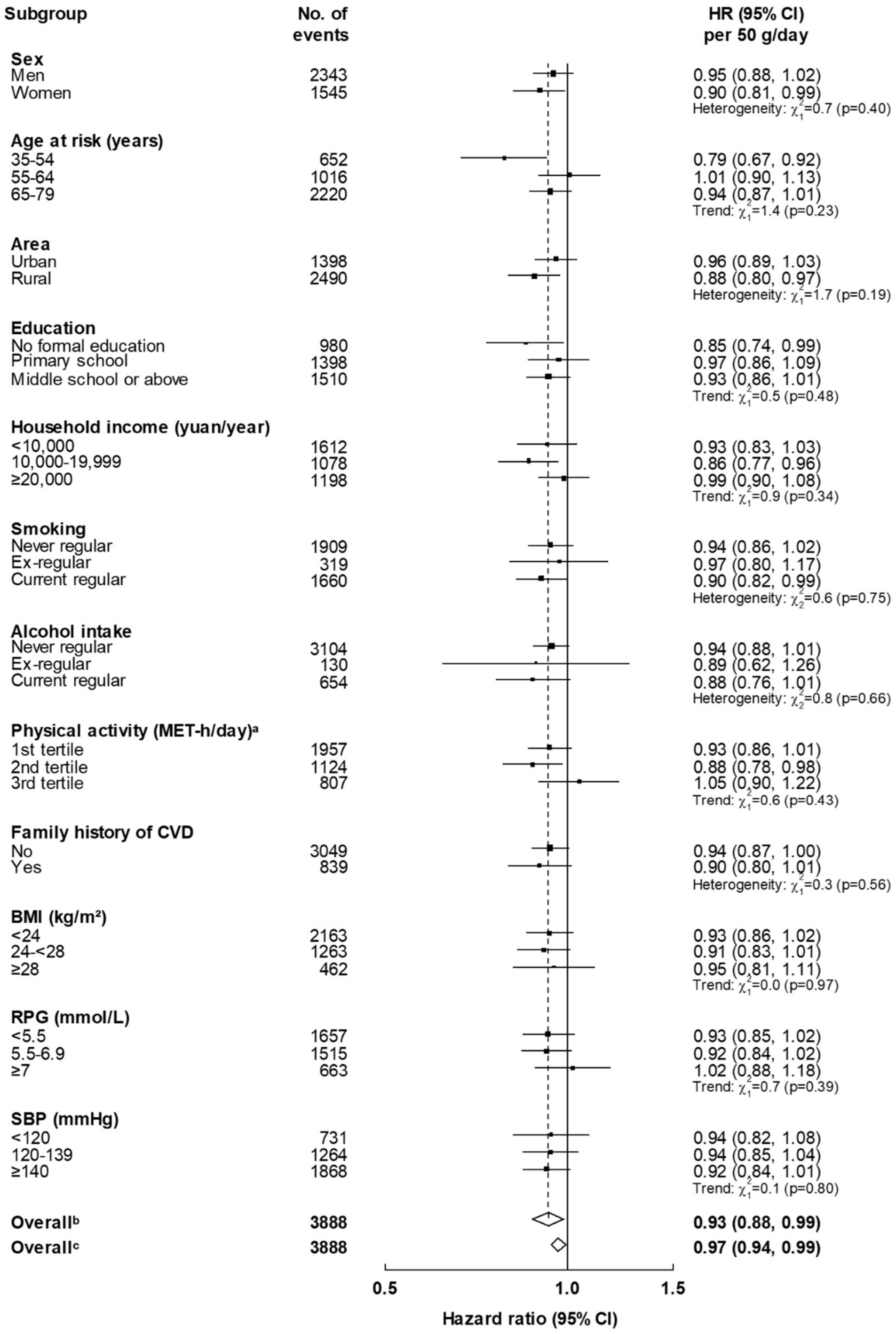

eFigure 11: Adjusted HRs (95% CIs) for ICH per 50 g/day of usual dairy intake by baseline characteristics. Events of ischemic heart disease (IHD), acute myocardial infarction (MI), intracerebral haemorrhage (ICH) and ischemic stroke (IS) were censored for each other. Analysis was stratified by age-at-risk (continuous variable), sex (dichotomous variable) and region (ten regions) and were adjusted for baseline age (continuous), education (four categories), income (four categories), smoking (four categories), alcohol consumption (four categories), total physical activity (continuous variable), family history of cardiovascular disease (CVD) (dichotomous variable), consumption of fresh fruit (five categories), red meat (four categories), poultry (three categories), fish (four categories) and eggs (four categories) and body mass index (BMI) (continuous). [a]Sex-specific physical activity assessed as metabolic equivalent of task (MET) (h/day). [b]Overall hazard ratio (HR) per 50 g/day usual dairy intake after correcting for regression dilution bias. [c]Overall HR per 50 g/day baseline dairy intake before correcting for regression dilution bias. Black squares, HRs (size is inversely proportional to the variance of the $\log_e$ of HR); horizontal lines represent 95% confidence intervals (CIs); white diamonds, overall HRs (95% CIs); 'No of events' refers to the number of incident CVD events or deaths in each group; the subscript numbers in the chi-square values represent the degrees of freedom.

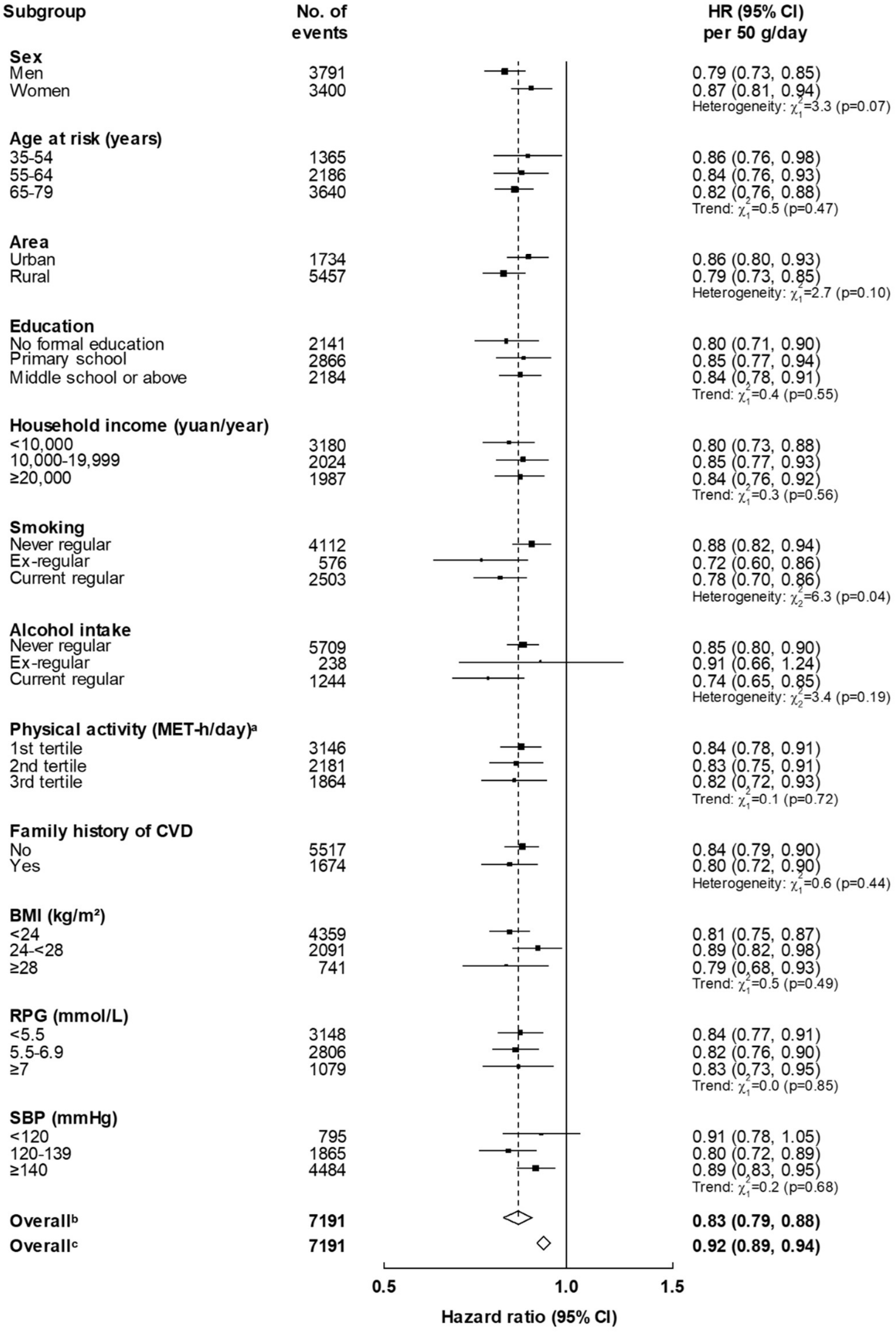

eFigure 12: Adjusted HRs (95% CIs) for cardiovascular death per 50 g/day of usual dairy intake by baseline characteristics. Analysis was stratified by age-at-risk (continuous variable), sex (dichotomous variable) and region (ten regions) and were adjusted for baseline age (continuous), education (four categories), income (four categories), smoking (four categories), alcohol consumption (four categories), total physical activity (continuous variable), family history of cardiovascular disease (CVD) (dichotomous variable), consumption of fresh fruit (five categories), red meat (four categories), poultry (three categories), fish (four categories) and eggs (four categories) and body mass index (BMI) (continuous). [a]Sex-specific physical activity assessed as metabolic equivalent of task (MET) (h/day). [b]Overall hazard ratio (HR) per 50 g/day usual dairy intake after correcting for regression dilution bias. [c]Overall HR per 50 g/day baseline dairy intake before correcting for regression dilution bias. Black squares, HRs (size is inversely proportional to the variance of the $\log_e$ of HR); horizontal lines represent 95% confidence intervals (CIs); white diamonds, overall HRs (95% CIs); 'No of events' refers to the number of incident CVD events or deaths in each group; the subscript numbers in the chi-square values represent the degrees of freedom.

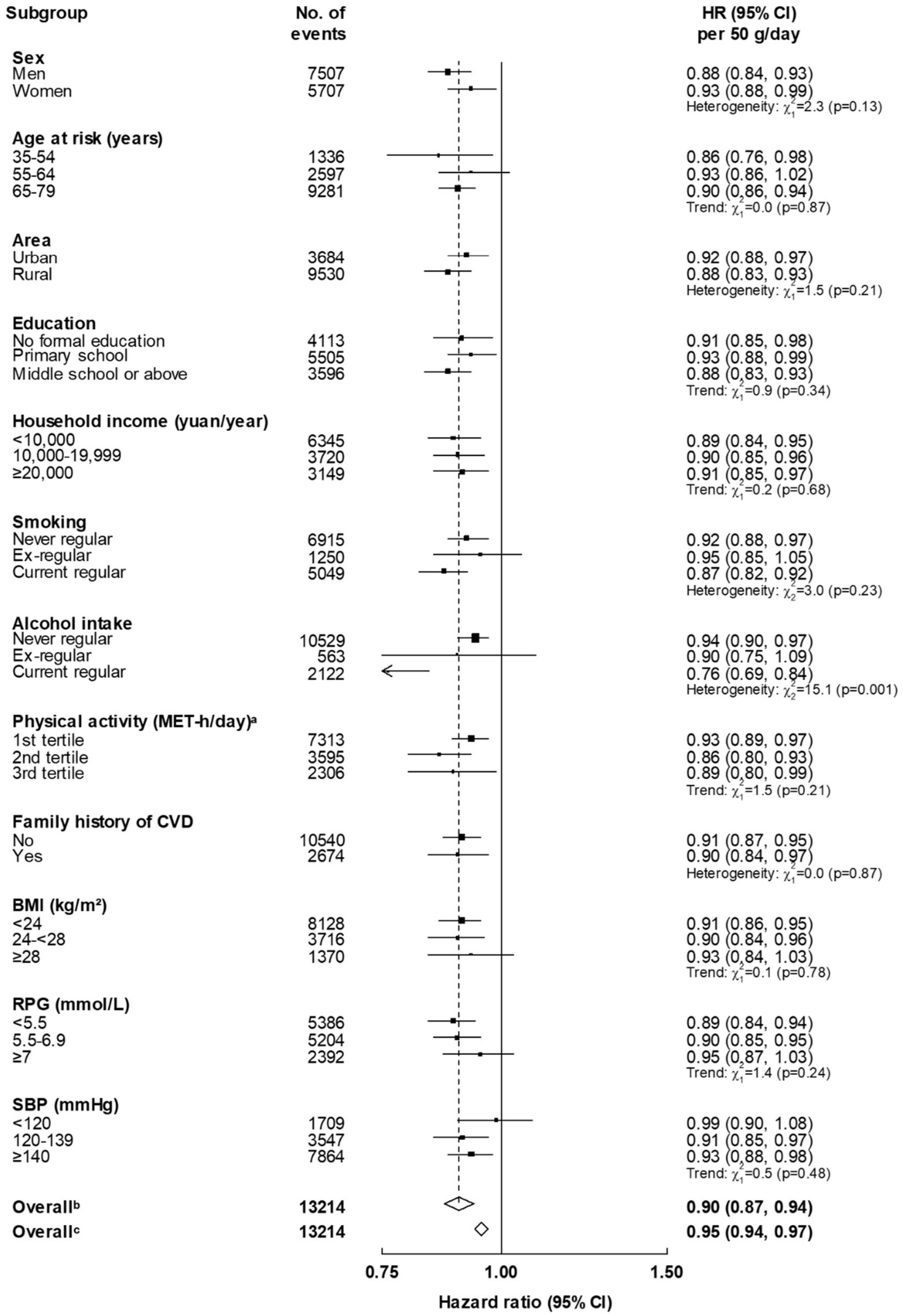

eFigure 13: Adjusted HRs (95% CIs) for IHD per 50 g/day of usual dairy intake by baseline characteristics. Events of ischemic heart disease (IHD), acute myocardial infarction (MI), intracerebral haemorrhage (ICH) and ischemic stroke (IS) were censored for each other. Analysis was stratified by age-at-risk (continuous variable), sex (dichotomous variable) and region (ten regions) and were adjusted for baseline age (continuous), education (four categories), income (four categories), smoking (four categories), alcohol consumption (four categories), total physical activity (continuous variable), family history of cardiovascular disease (CVD) (dichotomous variable), consumption of fresh fruit (five categories), red meat (four categories), poultry (three categories), fish (four categories) and eggs (four categories) and body mass index (BMI) (continuous). [a]Sex-specific physical activity assessed as metabolic equivalent of task (MET) (h/day). [b]Overall hazard ratio (HR) per 50 g/day usual dairy intake after correcting for regression dilution bias. [c]Overall HR per 50 g/day baseline dairy intake before correcting for regression dilution bias. Black squares, HRs (size is inversely proportional to the variance of the $\log_e$ of HR); horizontal lines represent 95% confidence intervals (CIs); white diamonds, overall HRs (95% CIs); 'No of events' refers to the number of incident CVD events or deaths in each group; the subscript numbers in the chi-square values represent the degrees of freedom.

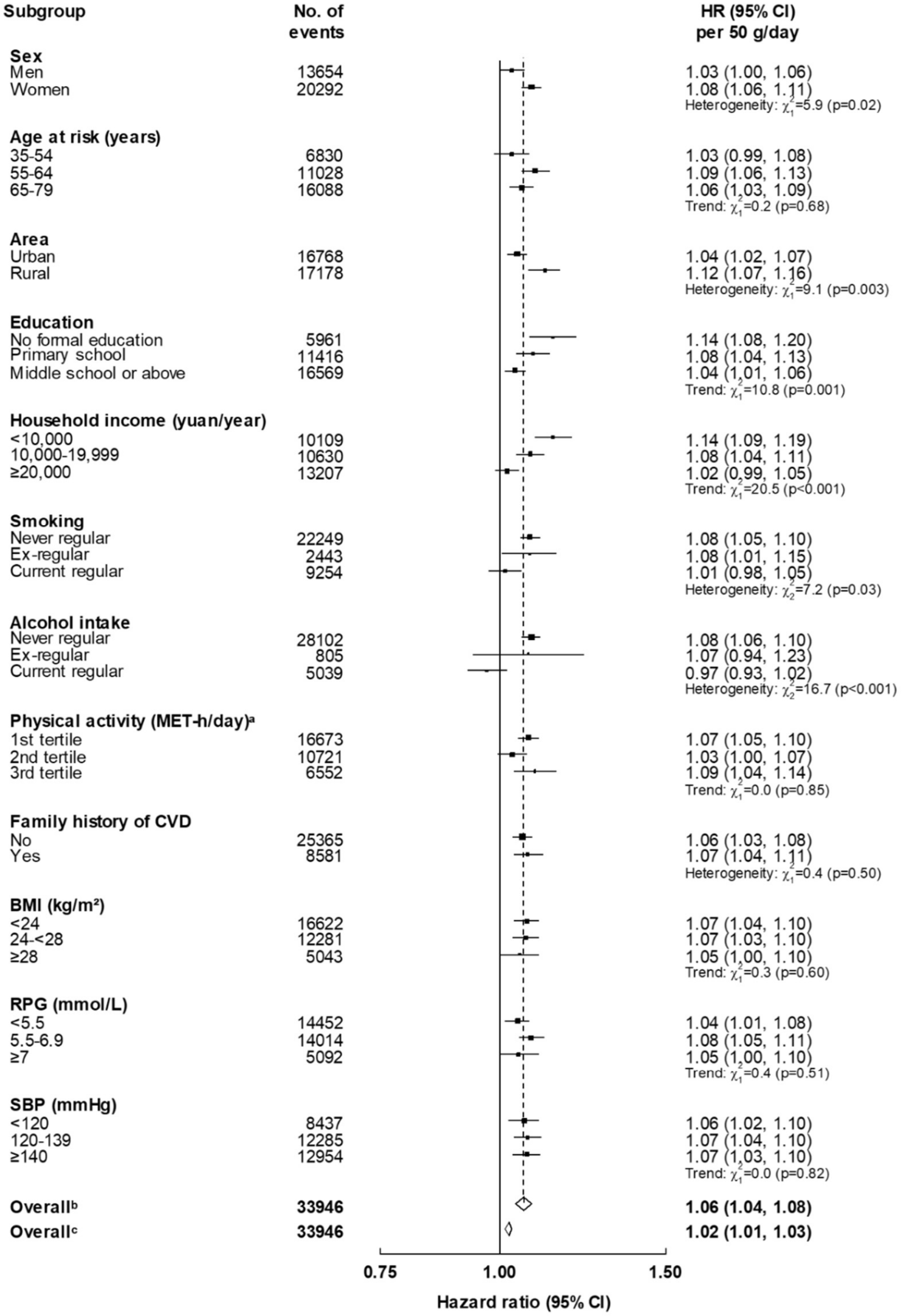

Supplement: Multimedia component 1 [file mmc1.pdf]
